# Reductions in Motor Unit Firing are Associated with Clinically Meaningful Leg Extensor Weakness in Older Adults

**DOI:** 10.1007/s00223-023-01123-x

**Published:** 2023-08-21

**Authors:** Nathan P. Wages, Mohamed H. Mousa, Leatha A. Clark, Dallin Tavoian, W. David Arnold, Sherif M. Elbasiouny, Brian C. Clark

**Affiliations:** 1 Ohio Musculoskeletal and Neurological Institute, Ohio University, 250 Irvine Hall, 1 Ohio University, Athens, OH 45701 USA; 2 Department of Biomedical Sciences, Ohio University, 250 Irvine Hall, 1 Ohio University, Athens, OH 45701 USA; 3 Department of Neuroscience, Cell Biology & Physiology, Wright State University, 350 NEC Building, 3640 Colonel Glenn Highway, Dayton, OH 45435 USA; 4 Department of Biomedical, Industrial & Human Factors Engineering, Wright State University, Dayton, OH USA; 5 Department of Family Medicine, Ohio University, Athens, OH USA; 6 Department of Physiology, University of Arizona, Tucson, AZ USA; 7 NextGen Precision Health, The University of Missouri, Columbia, MO USA; 8 Division of Geriatric Medicine, Ohio University, Athens, OH USA

**Keywords:** Dynapenia, Muscle strength, Motoneuron, Motor unit, Older adults, Sarcopenia

## Abstract

Weakness, one of the key characteristics of sarcopenia, is a significant risk factor for functional limitations and disability in older adults. It has long been suspected that reductions in motor unit firing rates (MUFRs) are one of the mechanistic causes of age-related weakness. However, prior work has not investigated the extent to which MUFR is associated with clinically meaningful weakness in older adults. Forty-three community-dwelling older adults (mean: 75.4 ± 7.4 years; 46.5% female) and 24 young adults (mean: 22.0 ± 1.8 years; 58.3% female) performed torque matching tasks at varying submaximal intensities with their non-dominant leg extensors. Decomposed surface electromyographic recordings were used to quantify MUFRs from the vastus lateralis muscle. Computational modeling was subsequently used to *independently* predict how slowed MUFRs would negatively impact strength in older adults. Bivariate correlations between MUFRs and indices of lean mass, voluntary activation, and physical function/mobility were also assessed in older adults. *Weak* older adults (*n* = 14) exhibited an approximate 1.5 and 3 Hz reduction in MUFR relative to *non-weak* older adults (*n* = 29) at 50% and 80% MVC, respectively. Older adults also exhibited an approximate 3 Hz reduction in MUFR relative to young adults at 80% MVC only. Our model predicted that a 3 Hz reduction in MUFR results in a strength decrement of 11–26%. Additionally, significant correlations were found between slower MUFRs and poorer neuromuscular quality, voluntary activation, chair rise time performance, and stair climb power (*r*’s = 0.31 to 0.43). These findings provide evidence that slowed MUFRs are mechanistically linked with clinically meaningful leg extensor weakness in older adults.

**Supplementary Information:**

The online version contains supplementary material available at 10.1007/s00223-023-01123-x.

## Introduction

More than 40% of older adults in the U.S. have at least one functional limitation while performing daily tasks considered essential for maintaining independence [1]. In fact, approximately 30% of older adult women and approximately 15% of older adult men report an inability to lift or carry 10 pounds [2]. Most notably, weakness, the key characteristic of the most recent sarcopenia definitions [3], has long been recognized as a major determinant of physical limitations and poor health in older adults [4–9]. Thus, the preservation of strength, and ultimately physical function/mobility, in older adults continues to be a major public health priority as it drastically reduces healthcare costs and improves quality of life [10].

The mechanisms of age-related weakness are multifactorial, with neurologic and skeletal muscle factors being key contributors to strength [11–15]. Regarding neural factors, skeletal muscle force is determined via recruitment of adequate numbers of alpha-motoneurons (MNs) and the modulation of their firing rates (i.e., rate coding). It has long been postulated that reductions in motor unit (a single MN and the muscle fibers it innervates) firing rate (MUFR) are one of the mechanistic causes of age-related weakness [16–22]. However, there are discrepancies in the literature regarding age-related changes in MUFR with some authors reporting slower MUFRs for older adults relative to young adults [18, 21–25], while others report similar MUFRs between older adults and young adults [17, 26–31]. For instance, Roos et al. [26] and Kirk et al. [31] reported that MUFRs were similar between older adults and young adults for the vastus medialis and gastrocnemii muscles, and Dalton et al. [27] reported that slower MUFRs were only observed in older adults during low-to-moderate contractions intensities (i.e., ≤ 50% of maximal strength) for the soleus muscle. In contrast, Connelly et al. [23], Kamen and Knight [21], Christie and Kamen [18], and Piasecki et al. [22] reported that older adults have slower MUFRs relative to young adults at all contraction intensities for the vastus lateralis and tibialis anterior muscles. These discrepancies likely arise for several reasons, ranging from differences in skeletal muscle group(s) examined, contraction intensities performed, participant sample sizes, instrumentation, analytical aspects of the motor unit recordings, etc. In addition, prior work has simply compared older adults to young adults, with little attention given to whether MUFRs differ between older adults with low muscle function (e.g., weakness) relative to those who are higher functioning.

In this work, we sought to use an interdisciplinary approach (human recordings and computational modeling) to investigate the extent to which MUFRs are associated with clinically meaningful weakness in older adults (weakness classifications were determined using the cut points suggested by Manini et al. [4] as part of the Health, Aging, and Body Composition Study). Specifically, the purpose of this study was to determine MUFRs between older adults with clinically meaningful leg extensor weakness [3, 4] relative to older adults without leg extensor weakness via decomposed surface electromyographic recordings. Our a priori hypothesis was that, for the vastus lateralis muscle, *weak* older adults would have slower MUFRs relative to *non-weak* older adults at moderate-to-high contraction intensities (i.e., ≥ 50% of maximal strength).

## Methods

A full, detailed description of the methodology, along with corresponding citations, are provided as supplemental material (insert link to supplemental material). Here, due to space limitations, we present an abbreviated overview of our methodology.

### General Overview

**Fig. 1:**
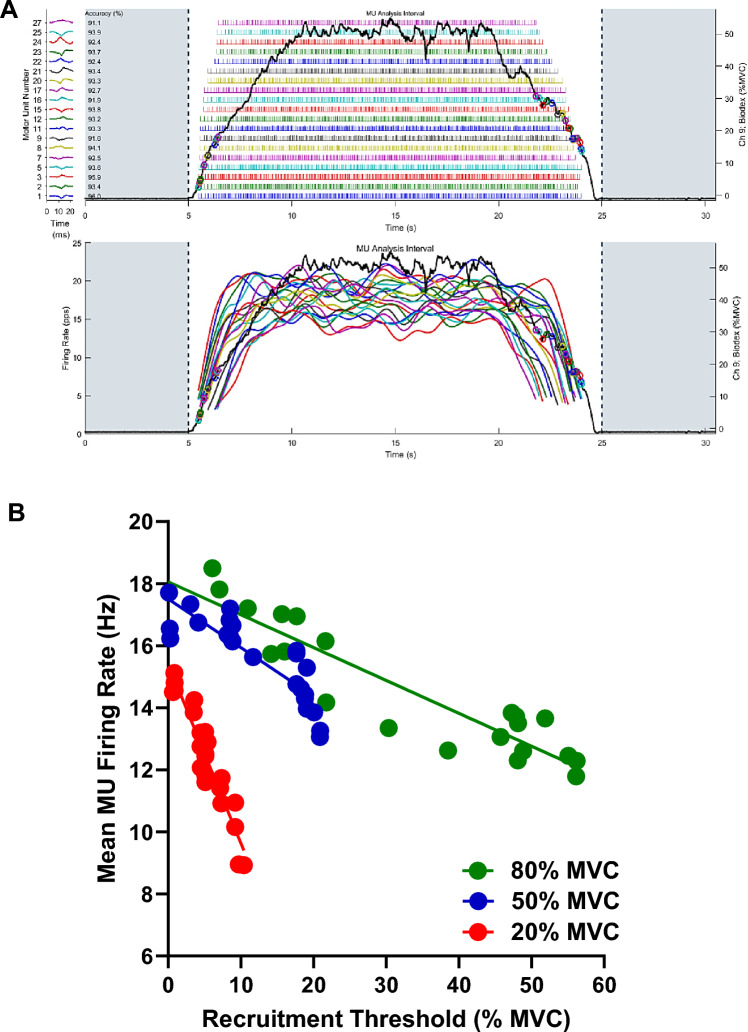
Example of motor unit firing rate profile (**a**) and linear regression of the motor unit firing rate vs. recruitment threshold plot used to calculate the *y*-intercept (**b**) for one older adult participant. **a** Torque-matching task and the identification of the associated motor units during a 50% MVC. Torque is in black and MUs are illustrated in color (e.g., circles on the torque trace indicate derecruitment time points and each colored line indicates a particular motor unit). *Top*: Individual motor unit firing rates underlie the target torque tracing. *Bottom*: Individual motor unit firing rates were smoothed by low-pass filtering each motor unit’s impulse train with a 1-s Hanning window. **b** The mean firing rate for each motor unit was plotted against its recruitment threshold at each contraction intensity (i.e., 20%, 50%, 80% MVC). Linear regression was applied to calculate the slope and *y*-intercept at each intensity level. To control for the inherent influence of larger-threshold motor units firing slower than lower-threshold motor units, we used the *y*-intercept value as our primary variable of interest (referred to as ‘normalized MUFR’ in the manuscript). *Hz* Hertz, *MU* motor unit, *MVC* maximal voluntary isometric contraction

Forty-three older adults and 24 young adults participated in this study. Participants had their non-dominant isokinetic and isometric leg extensor strength, as well as their handgrip strength, assessed. Participants also performed physical function/mobility tests, body composition assessments via dual-energy x-ray absorptiometry (DXA), and completed trapezoidal, target torque matching tasks at contraction intensities of 20%, 50%, and 80% of their maximal volitional contraction (MVC). Decomposed surface electromyographic (EMG) recordings were used to estimate MUFRs from the non-dominant vastus lateralis muscle during these target torque matching tasks. The primary outcome of interest was the y-intercept calculated from the mean MUFR versus the recruitment threshold scatterplot fit with a linear regression (calculated on a subject-by-subject basis at each contraction intensity; see Fig. 1). The y-intercept method, as opposed to using simply MUFRs at given contraction intensities, was preferred because it acts as a normalization process adjusting for the inherent influence of each motor unit’s recruitment threshold on its respective firing rate (see supplemental methods for further details). For clarity, henceforth the y-intercept data will be referred to as ‘normalized MUFR’. Subsequently, a multi-scale, high-fidelity, anatomically-detailed computational model was used to *independently* predict how reductions in normalized MUFR would negatively impact strength in older adults. Additionally, associations between normalized MUFRs and indices of lean mass, voluntary activation, and physical function/mobility were performed via bivariate correlations.

### Participants

Forty-three community-dwelling older adults (63–90 years, mean: 75.4 ± 7.4 years; 46.5% female) and 24 young adults (19–25 years, mean: 22.0 ± 1.8 years; 58.3% female) were included in the primary analysis (see Tables 1 and 2). Participants were living independently and were free of overt musculoskeletal and neurological disease. The Ohio University Institutional Review Board approved this study, and all study participants provided written informed consent in accordance with the Declaration of Helsinki.

**Table 1:** Descriptive characteristics of study population (mean ± SD)

	Age-related weakness			Older adult phenotypic weakness		
	Young adults *N* = 24	Older adults *N* = 43		Non-weak *n* = 29		Weak *n* = 14
Age (years)	22.0 ± 1.8	75.4 ± 7.4^a^		72.8 ± 6.4		80.9 ± 6.4^a^
Women (%)	58.3	46.5		44.8		50
Height (cm)	170.4 ± 8.8	166.1 ± 11.1^a^		167.6 ± 8.7		162.9 ± 14.7
Weight (kg)	71.3 ± 12.6	76.0 ± 15.9		75.0 ± 16.4		78.0 ± 15.2
Body mass Index (kg/m^2^)	24.3 ± 2.9	27.4 ± 4.8^a^		26.5 ± 4.8		29.3 ± 4.5
Body fat (%)	27.8 ± 7.1	33.1 ± 7.3^a^		31.7 ± 7.8		36.0 ± 5.5^a^
Thigh lean mass (kg)	5.6 ± 2.0	5.1 ± 1.0^a^		5.2 ± 1.1		4.9 ± 1.0
Appendicular lean mass/height^2^	7.4 ± 1.6	7.0 ± 1.2		7.1 ± 1.3		6.9 ± 1.2
Isometric LE strength (N-m)	156.1 ± 55.7	85.2 ± 36.0^a^		94.7 ± 36.0		65.5 ± 27.8^a^
Relative isokinetic LE strength (N-m/kg)	2.5 ± 0.4	1.3 ± 0.4^a^		1.5 ± 0.4		0.8 ± 0.2^a^
Neuromuscular quality (N-m/kg)	31.4 ± 4.4	19.0 ± 5.1^a^		21.6 ± 3.9		13.7 ± 2.7^a^
Handgrip strength (kg)	36.3 ± 10.8	27.9 ± 9.7^a^		30.1 ± 9.3		23.2 ± 9.1^a^
Moderate-to-vigorous activity (mins/week)	171.5 ± 51.9	112.5 ± 57.9^a^		125.5 ± 54.3		85.7 ± 57.5^a^
Chair rise time (s)	–	11.0 ± 3.5		9.4 ± 2.3		14.2 ± 3.5^a^
Stair climb power (watts)	415.2 ± 107.1	265.6 ± 96.7^a^		294.2 ± 93.0		206.3 ± 77.3^a^
Six-min walk gait speed (m/s)	–	1.4 ± 0.3		1.5 ± 0.2		1.1 ± 0.3^a^
Charlson index (% 10-year survival)	–	50.9 ± 21.7		54.8 ± 22.6		42.9 ± 17.9
SPPB score	–	11.0 ± 1.3		11.6 ± 0.7		9.8 ± 1.4^a^
RBANS score	102.5 ± 10.5	105.5 ± 13.0		107.2 ± 12.2		102.1 ± 14.5
Voluntary activation (%)	94.0 ± 3.5	89.5 ± 8.8^a^		90.8 ± 7.5		86.7 ± 10.7

For the voluntary activaiton mearement the sample sizes were *n* = 22 for young adults, *n* = 33 for older adults, *n* = 22 for non-weak older adults, and *n* = 11 for weak older adults

^a^Significant difference relative to the comparison group (e.g., young vs. older adults)

**Table 2:** Sex-specific descriptive characteristics of study population (mean ± SD)

	Age-related weakness				Older adult phenotypic weakness			
	Young adults *N* = 24		Older adults *N* = 43		Non-weak *n* = 29		Weak *n* = 14	
	Males *n* = 10	Females *n* = 14	Males *n* = 23	Females *n* = 20	Males *n* = 16	Females *n* = 13	Males *n* = 7	Females *n* = 7
Age (years)	22.9 ± 1.4	21.4 ± 1.8	75.4 ± 6.4^b^	75.4 ± 8.5^b^	73.5 ± 5.5	71.9 ± 7.4	79.9 ± 6.6^b^	81.9 ± 6.7^b^
Height (cm)	178.2 ± 6.9	164.9 ± 5.0^a^	173.6 ± 7.6	157.4 ± 7.5^a,b^	173.2 ± 6.6	160.8 ± 5.6^a^	174.7 ± 10.1	151.1 ± 6.6^a,b^
Weight (kg)	83.1 ± 6.9	62.9 ± 7.9^a^	84.1 ± 12.9	66.6 ± 14.1^a^	82.4 ± 13.5	65.8 ± 15.4^a^	88.0 ± 11.2	68.1 ± 12.1^a^
Body mass index (kg/m^2^)	26.0 ± 2.1	23.1 ± 2.8	27.9 ± 4.0	26.9 ± 5.7^b^	27.5 ± 4.4	25.3 ± 5.1	28.8 ± 2.8	29.9 ± 5.9^b^
Body fat (%)	22.0 ± 4.8	32.7 ± 4.4^a^	29.2 ± 5.7^b^	37.5 ± 6.5^a,b^	27.7 ± 6.0	36.6 ± 7.0^a^	32.8 ± 3.0	39.1 ± 5.8^a^
Thigh lean mass (kg)	7.2 ± 0.6	4.9 ± 0.7^a^	5.8 ± 0.7^b^	4.2 ± 0.6^a,b^	5.9 ± 0.7	4.3 ± 0.7^a^	5.6 ± 0.5	4.1 ± 0.5^a^
Appendicular lean mass/height^2^	8.8 ± 1.1	6.3 ± 0.8^a^	7.8 ± 0.8^b^	6.1 ± 1.0^a^	8.0 ± 0.8	6.0 ± 0.8^a^	7.5 ± 0.6	6.3 ± 1.3^a^
Isometric LE strength (N-m)	213.2 ± 34.0	115.3 ± 19.6^a^	109.6 ± 32.3^b^	63.5 ± 17.9^a,b^	118.6 ± 33.2	70.5 ± 17.4^a^	89.0 ± 18.9^b^	50.5 ± 10.2^a^
Relative isokinetic LE strength (N-m/kg)	2.8 ± 0.4	2.3 ± 0.3^a^	1.4 ± 0.5^b^	1.1 ± 0.4^a,b^	1.6 ± 0.4	1.3 ± 0.3^a^	0.9 ± 0.1^b^	0.8 ± 0.2^b^
Neuromuscular quality (N-m/kg)	32.6 ± 4.4	30.4 ± 4.3^a^	20.2 ± 5.4^b^	17.7 ± 4.6^a,b^	22.7 ± 4.2	20.3 ± 3.1	14.4 ± 2.8^b^	13.0 ± 2.7^b^
Handgrip strength (kg)	47.0 ± 4.6	27.4 ± 4.2^a^	34.2 ± 8.4^b^	20.6 ± 4.6^a,b^	35.8 ± 8.8	23.2 ± 2.9^a^	30.5 ± 6.7	15.9 ± 3.3^a,b^
Moderate-to-vigorous activity (mins/week)	133.3 ± 70.4	202.7 ± 193.6	120.8 ± 56.5	103.1 ± 59.4^b^	124.6 ± 52.8	126.6 ± 58.2	112.0 ± 67.9	59.4 ± 30.9^b^
Chair rise time (s)	–	–	10.6 ± 2.3	11.4 ± 4.6^a^	10.0 ± 2.1	8.7 ± 2.4	12.2 ± 1.8^b^	16.2 ± 3.7^a,b^
Stair climb power (watts)	506.5 ± 72.8	339.1 ± 60.5^a^	313.2 ± 84.0^b^	210.8 ± 81.4^a,b^	330.6 ± 93.0	249.4 ± 73.7^a^	273.5 ± 39.3	139.0 ± 29.1^a,b^
Six-min walk gait speed (m/s)	–	–	1.4 ± 0.2	1.2 ± 0.4^a^	1.5 ± 0.2	1.5 ± 0.2	1.3 ± 0.2	0.8 ± 0.2^a,b^
Charlson index (% 10-year survival)	–	–	50.0 ± 22.3	51.9 ± 21.5	55.7 ± 20.7	53.6 ± 25.5	36.9 ± 21.5	48.8 ± 12.1
SPPB score	–	–	11.3 ± 1.0	10.7 ± 1.6	11.5 ± 0.9	11.7 ± 0.5	10.7 ± 1.0^b^	8.9 ± 1.1^a,b^
RBANS score	102.9 ± 15.9	102.2 ± 4.5	102.6 ± 13.2	108.8 ± 12.3	104.1 ± 11.7	110.8 ± 12.2	99.1 ± 16.7	105.1 ± 12.5
Voluntary activation (%)	92.7 ± 3.4	94.8 ± 3.4	88.7 ± 9.7	90.1 ± 8.0^b^	90.1 ± 7.5	91.4 ± 7.8	86.4 ± 13.1	87.0 ± 8.6

For the voluntary activaiton mearement the sample sizes were *n* = 22 for young adults (8 males and 14 females), *n* = 33 for older adults (16 males and 17 females), *n* = 22 for non-weak older adults (10 males and 12 females), and *n* = 11 for weak older adults (6 males and 5 females)

^a^Significant difference relative to the sex-specific comparison within sub-groups (e.g., young adult males vs. females)

^b^Significant difference relative to the sex-specific comparison between groups (e.g., young vs. older adult males)

To characterize the older adults, we measured their (1) six-minute walk gait speed, (2) short physical performance battery test, and (3) comorbidities via the Charlson Comorbidity Index. In all participants, we assessed body composition (including appendicular and thigh lean mass) via DXA (Hologic Discovery QDR model Series, Waltham, MA, USA), time of moderate-to-vigorous intensity physical activity via accelerometry (ActiGraph, wgt3x-bt, Pensacola, FL), and neuropsychological status via the Repeatable Battery for the Assessment of Neuropsychological Status (RBANS). See Tables 1 and 2.

### Leg Extension and Handgrip Strength

Non-dominant, isometric and isokinetic leg extension strength measures were recorded via a dynamometer (Biodex System 4 Dynamometer, Biodex Medical Systems Inc., Shirley, NY). Maximal handgrip strength was assessed using a portable JAMAR® Hydraulic Hand Dynamometer; (Model 5030 J1; Lafayette Instrument Co.; Lafayette, Indiana).

### Clinically Meaningful Weakness Classifications

As stated above, older adult clinically meaningful weakness phenotypes were determined using the same isokinetic leg extension strength protocol that was performed in the Health, Aging, and Body Composition Study from which the leg extensor weakness thresholds were derived [4]. Specifically, older adults were classified as ‘weak’ for the leg extensors if their non-dominant isokinetic (60°/sec) strength relative to their body weight was ≤ 1.12 Nm/kg for men and ≤ 1.00 Nm/kg for women. This weakness classification represents the 1st & 3rd decile (for men & women, respectively) of sex-specific, relative strength values, which has been shown to be predictive of non-disabled older adults subsequently developing severe mobility limitations [4]. These cut points were derived from a cohort of 1355 men and 1429 women (mean: 73.6 ± 2.85 years) that were tracked over ~ 5.9 years [4]. Older adults with values above these sex-specific cut points were classified as ‘non-weak’. Of note, all young adults were classified as ‘non-weak’ as their values were above the sex-specific 1st & 3rd decile cut points. All assessments conducted were performed by lab personnel blinded to weakness classification.

### Neuromuscular Quality

Isokinetic leg extensor strength of the non-dominant leg was expressed relative to thigh lean mass, obtained via whole body DXA scans (Hologic Discovery QDR model Series, Waltham, MA, USA). Calculation of whole-body, appendicular, and non-dominant thigh lean tissue mass were performed using the analysis package (Hologic APEX, ver. 4.0.2).

### Voluntary Activation

Here, the doublet interpolation technique was performed. Specifically, the participant was asked to perform one-to-two 5-s isometric MVCs while a 100-Hz supramaximal doublet was delivered during the peak force output followed by a second doublet delivered to the resting muscle. The increase in force immediately following the stimulation was expressed relative to a potentiated response evoked by the same doublet applied at rest (i.e., one-to-two seconds after the supramaximal doublet stimulation during the MVC). We should also mention that 10 older adults and two young adults were unable to complete the voluntary activation testing due to discomfort associated with the stimulation.

### Motor Unit Recordings and Analyses

Trapezoidal, isometric contractions were performed using the same mechanical setup as described for the leg extension strength measures at 20%, 50%, and 80% MVC. Target torque matching templates contained, in the following order, a(n) (1) quiescent period (4 s), (2) ascending phase (10% MVC/s), (3) steady-state, plateau phase (10 s for the 20% and 50% MVC, and 8 s for the 80% MVC), (4) descending phase (10% MVC/sec), and (5) additional quiescent period (4 s). All torque matching templates were completed twice with 1 min of rest between bouts.

Surface EMG signals were recorded from the non-dominant vastus lateralis muscle via a Bagnoli Desktop system (Delsys, Inc., Natick, MA). Signals were detected with a 5-pin array EMG sensor (Delsys, Inc., Natick, MA). Filtered EMG signals served as the input to the Precision Decomposition III algorithm, which was utilized via dEMG Analysis software (ver. 1.1, Delsys, Inc., Natick, MA). Decomposition-Synthesize-Decomposition-Compare testing was used to remove motor units with detection accuracy < 90.0%. Before analysis, all MUFR curves were smoothed by low-pass filtering each motor unit’s impulse train with a 1-s Hanning window.

The mean MUFRs data was individually normalized at each contraction intensity (see supplemental material for further details). Here, the y-intercept from the mean MUFR versus recruitment threshold scatterplot fit with a linear regression was used as this approach theoretically controls for the ‘onion skin phenomenon’ of MUFRs (i.e., larger-threshold motor units fire slower than lower-threshold motor units). Specifically, for each participant at each contraction intensity (20%, 50%, and 80% MVC) the coefficients of the linear regression (y-intercept and slope) were calculated for the mean MUFRs versus the recruitment threshold scatterplot. Thus, the y-intercepts (our index for ‘normalized MUFR’) and slopes were determined for each participant in a particular group, at each contraction intensity, before averaging the aggregated data by group and subsequently disaggregating the data by sex.

### Computational Modeling

Developed with NEURON simulation environment (ver. 7.6). Simulations were run on the Neuroscience Gateway Stampede2 KNL Super-computer with NEURON 7.6 tool. Analysis was performed in *MATLAB* (ver. 9.9.0 [R2020b]). For the vastus lateralis computer model employed herein, we incorporated several features of biological variability (i.e., heterogeneity and overlap in cell properties of different MN types), which simulates MN recruitment, firing rates, and force generation more accurately than computer models missing those features. *See below for futher details.*

#### Vastus Lateralis Motoneuron Pool Model—Individual Cells

Cat MNs, as opposed to rodents, were used as the foundation of our human cell models as their electrical and firing properties, as well as force production, are very similar to humans. Specifically, the reconstructed morphologies of identified slow (S), fatigue-resistant (FR), and fast-fatiguing (FF) cat cells were used to represent the three-dimensional anatomy of model MNs (Fig. 4a). To mimic the physiological activation of MNs via synaptic inputs, excitatory synapses were distributed over the dendrites of each model cell following the realistic distribution of Ia afferent-to-motoneuron contacts labeled intracellularly with horseradish peroxidase in type-identified cat MNs. Additionally, the model MNs include somatic and dendritic voltage-gated and calcium-activated ion channels that mediate transient and persistent inward/outward currents underlying nonlinear MN firing properties. Moreover, their membrane electrical passive and active properties matched those measured experimentally from cat MN (see Fig. 5a).

#### Vastus Lateralis Motoneuron Pool Model—Synaptic Input

Individual cells in the MN pool model were stimulated through synaptic inputs with trapezoidal activation waveforms, like in our human experimental protocols. The conductance of these synaptic inputs was determined using effective synaptic currents of pyramidal inputs to spinal MNs, in which large MNs received higher synaptic current than small MNs (Fig. 5d). The mean MUFR of recruited motor units was measured in the simulation via a 200 ms moving window (during the plateau phase of trapezoidal waveforms).

#### Vastus Lateralis Motoneuron Pool Model—Motor Unit Type Composition

The percentage of different cell types (S, FR, FF) in the vastus lateralis muscle MN pool was determined using previously published older adult fiber type data (see supplemental material for further details). Fifty-eight percent of motor units were identified as Type I (S), 25% were Type IIA (FR), and 17% were Type IIB (FF). The average innervation ratio was estimated to be: 1 (Type I): 2.5 (Type IIA): 5 (Type IIB). Thus, the innervation ratio for Type IIA is 2.5 times greater than the innervation ratio for Type I, while the innervation ratio for Type IIB is 5 times greater than the innervation ratio with Type I. Based on these muscle fiber types and innervation ratio data, the MN pool innervating the vastus lateralis muscle for older adults consisted of ~ 81% S-type, ~ 14% FR-type, and ~ 5% FF-type cells. As the vastus lateralis muscle MN pool was modeled with 189 model cells in the present study, 153 were S-type, 27 were FR-type, and 9 were FF-type cells (Fig. 4a).

#### Vastus Lateralis Motoneuron Pool Model—Force Generation

Here, spike trains were converted to twitch forces in each cell in the MN pool model, and all motor unit forces were summated to simulate the total vastus lateralis muscle force (Fig. 5a). To simulate the changes in force production with aging, we adjusted the motor unit force parameters (twitch force amplitude and duration) by the percent changes observed from young adults to older adults (i.e., twitch force amplitude was decreased by 36% and twitch force duration was increased by 15%). Then, the synaptic drive to the MNs was reduced to record the change in predicted strength (Fig. 5b, secondary y-axis).

### Statistical Analysis

Repeated measures analysis of covariances (ANCOVAs) were performed to examine group differences in the respective dependent variable (e.g., normalized MUFR). Here, group (e.g., *weak* versus *non-weak* older adults) was a between-participant factor and contraction intensity were a within-participant factor (3 levels). Sidak post-hoc analyses were performed if a significant main effect of interaction was observed. One-way ANCOVAs were used for group wise comparisons. Sex was covaried in all aggregated data analyses. Additionally, directionally hypothesized bivariate correlations (i.e., one-tailed tests) were used to examine whether there were associations between slower normalized MUFRs and poorer neuromuscular quality, lower voluntary activation, and with reduced physical function/mobility in older adults. A p-value of ≤ 0.05 was required for statistical significance. Statistical Package for the Social Sciences (SPSS; version 25.0, Chicago, IL) was used for data analysis and presented as estimated marginal means ± the standard error of mean (SEM). Effect sizes (*ƞ*^2^) are also reported to aid in interpretation.

## Results

### ‘Weak’ versus ‘Non-Weak’ Older Adults

Older adult weakness phenotype group data was first aggregated for *weak* relative to *non-weak* older adults, covarying for sex. Next, this group data was disaggregated by sex to investigate sex-specific differences among and between weakness phenotypes. Of note, the disaggregated sex-specific data is likely underpowered, and thus should be interpreted with caution. These data are presented in Tables 3 and 4.

**Table 3:** Mean and normalized (linear regression) motor unit firing rates of study population (EMM ± SEM)

	Age-related weakness		Older adult phenotypic weakness	
	Young adults *N* = 24	Older adults *N* = 43	Non-weak *n* = 29	Weak *n* = 14
20% MVC				
Motor unit number	14 ± 1.0	13.2 ± 0.7	12.9 ± 0.9	14.4 ± 1.3
Slope	− 0.70 ± 0.25	− 1.11 ± 0.19	− 1.2 ± 0.3	− 0.95 ± 0.4
Y-intercept (Hz)	19.5 ± 0.6	19.3 ± 0.5	19.5 ± 0.6	19 ± 0.9
R2 (%)	66.2 ± 4.3	57.7 ± 3.2	54.6 ± 4.1	64.6 ± 5.8
50% MVC				
Motor unit number	21.7 ± 1.2	15.6 ± 0.9^a^	14.4 ± 1.1	18.5 ± 1.5
Slope	− 0.38 ± 0.06	− 0.47 ± 0.05	− 0.5 ± 0.07	− 0.42 ± 0.1
Y-intercept (Hz)	22.4 ± 0.5	21.6 ± 0.4	22.1 ± 0.4	20.5 ± 0.6^a^
R2 (%)	76.4 ± 3.9	70.0 ± 2.9	70.5 ± 3.5	69.1 ± 5.0
80% MVC				
Motor unit number	18.9 ± 1.3	13.7 ± 1.0^a^	13.5 ± 1.1	14.3 ± 1.6
S lope	− 0.29 ± 0.03	− 0.31 ± 0.02	− 0.33 ± 0.03	− 0.27 ± 0.05
(Hz)	25.5 ± 0.8	22.7 ± 0.6^a^	23.7 ± 0.7	20.6 ± 0.9^a^
R2 (%)	79.3 ± 3.6	71.3 ± 2.7	70.7 ± 3.8	72.3 ± 5.5

*EMM* estimated marginal means, *Hz * Hertz, *MVC* maximal voluntary isometric contraction, *SEM* standard error of the mean

^a^Significant difference relative to the comparison group (e.g., young vs. older adults)

**Table 4:** Sex-specific mean and normalized (linear regression) motor unit firing rates of study population (EMM ± SEM)

	Age-related weakness				Older adult phenotypic weakness			
	Young adults *N* = 24		Older adults *N* = 43		Non-weak *n* = 29		Weak *n* = 14	
	Males *n* = 10	Females *n* = 14	Males *n* = 23	Females *n* = 20	Males *n* = 16	Females *n* = 13	Males *n* = 7	Females *n* = 7
20% MVC								
Motor unit number	14.3 ± 1.7	13.2 ± 1.1	15.9 ± 1.1	10.5 ± 0.9^a^	15.7 ± 1.3	9.6 ± 1.1^a^	16.3 ± 2.0	12.1 ± 1.6
Slope	− 0.67 ± 0.37	− 0.70 ± 0.34	− 1.21 ± 0.25	− 1.01 ± 0.28	− 1.48 ± 0.3	− 0.85 ± 0.44	− 0.59 ± 0.51	− 1.29 ± 0.60
Y-intercept (Hz)	19.4 ± 1.1	19.4 ± 0.8	20.0 ± 0.7	18.6 ± 0.7	20.6 ± 0.6	18.2 ± 0.9^a^	18.6 ± 1.2	19.3 ± 1.2
R2 (%)	59.4 ± 6.2	70.5 ± 6.5	63.5 ± 4.1	51.4 ± 4.6^a,^^b^	58.7 ± 4.9	50.0 ± 6.7	74.4 ± 7.4	53.9 ± 9.2
50% MVC								
Motor unit number	22.4 ± 1.9	20.9 ± 1.7	17.1 ± 1.2^b^	14.2 ± 1.4^b^	15.4 ± 1.3	13.3 ± 1.8	21.0 ± 2.0^b^	15.7 ± 2.4
Slope	− 0.40 ± 0.13	− 0.35 ± 0.05	− 0.55 ± 0.08	− 0.37 ± 0.04^a^	− 0.61 ± 0.11	− 0.35 ± 0.06^a^	− 0.42 ± 0.17	− 0.40 ± 0.08
Y-intercept (Hz)	22.2 ± 0.8	22.5 ± 0.6	21.4 ± 0.5	21.7 ± 0.5	22.2 ± 0.5	21.7 ± 0.6	19.6 ± 0.8^b^	21.5 ± 0.8
R2 (%)	77.6 ± 3.9	75.0 ± 6.5	72.1 ± 2.5	67.7 ± 5.4	71.3 ± 3.1	69.7 ± 6.7	74.0 ± 4.6	63.9 ± 9.20
80% MVC								
Motor unit number	19.0 ± 1.9	18.6 ± 1.7	15.1 ± 1.3	12.2 ± 1.5^b^	15.7 ± 1.5	10.9 ± 1.6^a^	13.9 ± 2.3	14.8 ± 2.1
Slope	− 0.30 ± 0.05	− 0.28 ± 0.04	− 0.32 ± 0.04	− 0.31 ± 0.03	− 0.37 ± 0.04	− 0.30 ± 0.05	− 0.21 ± 0.06	− 0.33 ± 0.06
Y-intercept (Hz)	26.5 ± 1.5	24.7 ± 0.9	23.0 ± 1.0^b^	22.3 ± 0.7	24.2 ± 1.0	23.1 ± 0.8	20.3 ± 1.6^b^	20.7 ± 1.0
R2 (%)	77.8 ± 5.9	80.5 ± 4.4	71.3 ± 3.9	71.2 ± 3.7	73.6 ± 5.2	67.2 ± 5.6	66.1 ± 7.8	78.5 ± 7.6

*EMM* estimated marginal means, *Hz *Hertz, *MVC * maximal voluntary isometric contraction, *SEM* standard error of the mean

^a^Significant difference relative to the sex-specific comparison within sub-groups (e.g., young adult males vs. females)

^b^Significant difference relative to the sex-specific comparison between groups (e.g., young vs. older adult males)

#### Aggregated Group Data by Older Adult Weakness Phenotypes

**Fig. 2:**
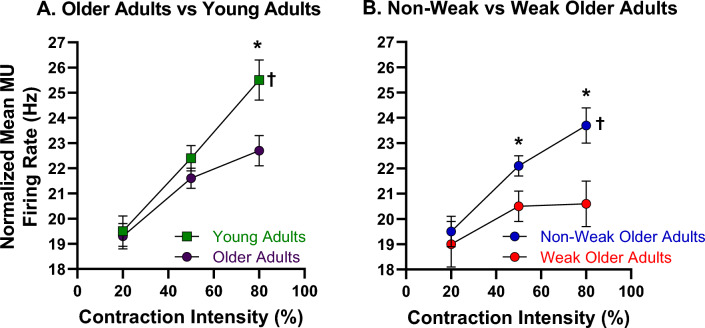
Older adults with clinically meaningful leg extensor weakness exhibit slowing in motor unit firing rates during higher intensity contractions. During moderate-to-high contraction intensities (i.e., ≥ 50% MVC) normalized motor unit firing rates were lower in older adults (purple circle; **a**) relative to young adults (green squares; **a**), as well as in older adults with clinically meaningful leg extensor weakness (*weak*- red circles; *non-weak*- blue circles; **b**). Asterisks and daggers represent significant differences between groups and intensities, respectively. *Hz * Hertz

When compared to *non-weak* older adults, *weak* older adults exhibited 1.6 and 3.1 Hz slower (7.2% and 13.1% slower, respectively) normalized MUFRs at 50% and 80% MVCs, respectively (22.1 vs. 20.5 Hz and 23.7 vs. 20.6 Hz, respectively). Specifically, a significant group by contraction intensity interaction was observed for normalized MUFR (Fig. 2; *p* = 0.05; *ƞ*^2^ = 0.07). Post-hoc analyses indicated that *non-weak* older adults demonstrated a sequential increase in normalized MUFR at each contraction intensity level (*p*’s = 0.01 to 0.03), whereas *weak* older adults did not exhibit any difference between contraction intensities (*p*’s = 0.09 to 1.0). Additionally, *weak* older adults exhibited slower normalized MUFRs relative to *non-weak* older adults at 50% and 80% MVCs (*p*’s = 0.03 and 0.01, respectively), but not at 20% MVC (*p* = 0.65). Moreover, a group by contraction intensity interaction was not observed for the linear regression slope (*p* = 0.88; *ƞ*^2^ < 0.01), nor for a group (*p* = 0.45; *ƞ*^2^ = 0.02), or a contraction intensity main effect (*p* = 0.21; *ƞ*^2^ = 0.04). These data are presented in Table 3 and Fig. 2b.

#### Disaggregated Sex-Specific Group Data by Weakness Phenotypes

When compared to *non-weak* older adult males, *weak* older adult males exhibited 2.6 and 3.5 Hz slower (11.7% and 16.1% slower, respectively) normalized MUFRs at 50% and 80% MVCs (22.2 vs. 19.6 Hz and 24.2 vs. 20.3 Hz, respectively). Specifically, a significant group by contraction intensity interaction was observed for normalized MUFR (*p* = 0.05; *ƞ*^2^ = 0.07). Post-hoc analysis indicated that *non-weak* older adult males demonstrated a sequential increase in normalized MUFR at each contraction intensity level (*p*’s < 0.01 to 0.04), whereas *weak* older adult males did not exhibit any difference between contraction intensities (*p*’s = 0.07 to 1.0). Additionally, *weak* older adult males exhibited slower normalized MUFRs relative to *non-weak* older adult males at 50% and 80% MVCs (*p*’s = 0.03 and 0.01, respectively), but not at 20% MVC (*p* = 0.68).

When compared to *non-weak* older adult females, *weak* older adult females did not exhibit any differences at any contraction intensity level (*p* = 0.08; *ƞ*^2^ = 0.13). Moreover, when compared to *non-weak* older adult males, *non-weak* older adult females exhibited 2.4 Hz slower (i.e., 11.7% slower; 20.6 vs. 18.2 Hz) normalized MUFRs at 20% MVC (*p* = 0.05), but not at 50% and 80% MVCs (*p*’s = 0.68 and *p* = 0.41, respectively). When *weak* older adult males and females were compared, there were no differences at any contraction intensity level (*p*’s = 0.11 to 0.82). Subsequently, a sex by weakness by contraction intensity interaction was not observed for the linear regression slope (*p* = 0.51; *ƞ*^2^ = 0.04), nor for sex by weakness (*p* = 0.95; *ƞ*^2^ < 0.01) or for sex by contraction intensity (*p* = 0.49; *ƞ*^2^ = 0.04). Furthermore, neither a main effect for weakness (*p* = 0.45; *ƞ*^2^ = 0.02) nor sex (*p* = 0.43; *ƞ*^2^ = 0.02) was observed. These data are presented in Table 4.

### Older Adults Versus Young Adults

Age-related group data was first aggregated for older adults relative to young adults, covarying for sex. Next, this group data was disaggregated by sex to investigate sex-specific differences among and between age. These data are presented in Tables 3 and 4.

#### Aggregated Group Data by Age

When compared to young adults, older adults exhibited 2.8 Hz slower (11% slower) normalized MUFRs at 80% MVC (25.5 vs. 22.7 Hz, respectively). Specifically, a significant group by contraction intensity interaction was observed for normalized MUFR (Fig. 1; *p* = 0.01; *ƞ*^2^ = 0.07). Post-hoc analyses indicated that young adults demonstrated a sequential increase in normalized MUFR at each contraction intensity level (*p*’s < 0.01), whereas older adults exhibited an increase between 20 and 50% MVCs (*p* < 0.01), and 20% and 80% MVCs (*p* < 0.01), but no difference between 50 and 80% MVCs (*p* = 0.20). Additionally, older adults exhibited slower normalized MUFRs relative to young adults at 80% MVC (*p* < 0.01), but not at 20% and 50% MVCs (*p*’s = 0.82 and 0.20, respectively). Moreover, a group by contraction intensity interaction was not observed for the linear regression slope (*p* = 0.27; *ƞ*^2^ = 0.02), nor for a group (*p* = 0.13; *ƞ*^2^ = 0.04), or a contraction intensity main effect (*p* = 0.17; *ƞ*^2^ = 0.03). These data are presented in Table 3 and Fig. 2a.

#### Disaggregated Sex-Specific Group Data by Age

When compared to young adult males, older adult males exhibited 3.5 Hz slower (13.2% slower) normalized MUFRs at 80% MVC (26.5 vs. 23.0 Hz, respectively). Specifically, a significant age by contraction intensity interaction was observed for normalized MUFR (*p* = 0.02; *ƞ*^2^ = 0.12). Post-hoc analysis indicated that young adult males demonstrated a sequential increase in normalized MUFR at each contraction intensity level (*p*’s < 0.01), whereas older adult males exhibited an increase between 20 and 50% MVCs (*p* < 0.01), and 20% and 80% MVCs (*p* < 0.01), but no difference between 50 and 80% MVCs (*p* = 0.19). Additionally, older adult males exhibited slower normalized MUFRs relative to young adult males at 80% MVC (*p* = 0.03), but not at 20% and 50% MVCs (*p* = 0.66 and *p* = 0.41, respectively).

When compared to young adult females, older adult females did not exhibit any differences at any contraction intensity level (*p* = 0.32; *ƞ*^2^ = 0.04). Moreover, when young adult males and females were compared, there were no differences at any contraction intensity level (*p*’s = 0.31 to 0.95). Similarly, when older adult males and females were compared, there were no differences at any contraction intensity (*p*’s = 0.15 to 0.66). Subsequently, a sex by age by contraction intensity interaction was not observed for the linear regression slope (*p* = 0.89; *ƞ*^2^ < 0.01), nor for sex by intensity (*p* = 0.93; *ƞ*^2^ < 0.01), or for age by contraction intensity (*p* = 0.27; *ƞ*^2^ = 0.02). Furthermore, neither a main effect for age (*p* = 0.12; *ƞ*^2^ = 0.04) nor sex (*p* = 0.51; *ƞ*^2^ < 0.01) was observed. These data are presented in Table 4.

### Association Between Normalized Motor Unit Firing Rates and Indices of Muscle/Physical Function

**Fig. 3:**
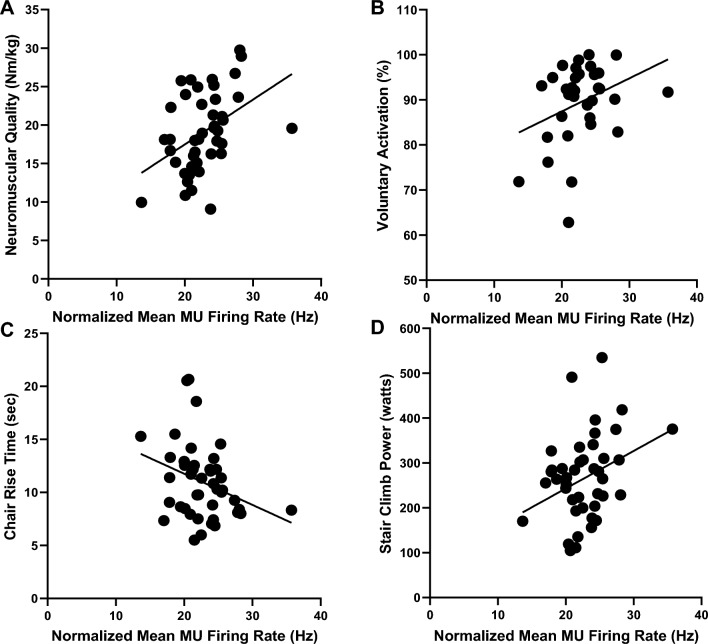
Slower motor unit firing rates at 80% MVC is associated with poorer neuromuscular quality, lower voluntary activation, and reduced physical function/mobility in older adults**.** Normalized motor unit firing rates at 80% MVC were associated with neuromuscular quality (**a** [top left]; *r* = 0.43; *p* < 0.01), voluntary activation (**b** [top right]; *r* = 0.33; *p* = 0.03), chair rise time (**c** [bottom left]; *r* = 0.31; *p* = 0.02), and stair climb power (**d** [bottom right]; *r* = 0.33; *p* = 0.015). *Hz *Hertz, *MVC *maximum voluntary isometric contraction, *MU* motor unit

The data presented herein is limited to older adults at 80% MVC. Significant associations were observed between slower normalized MUFRs and poorer neuromuscular quality (*r* = 0.43; *p* < 0.01), as well as lower voluntary activation (*r* = 0.33; *p* = 0.029). Additionally, significant associations were observed between slower normalized MUFRs and reduced chair rise time performance (*r* = 0.31; *p* = 0.020), as well as diminished stair climb power (*r* = 0.33; *p* = 0.015). These data are presented in Fig. 3.

### Computational Modeling

**Fig. 4:**
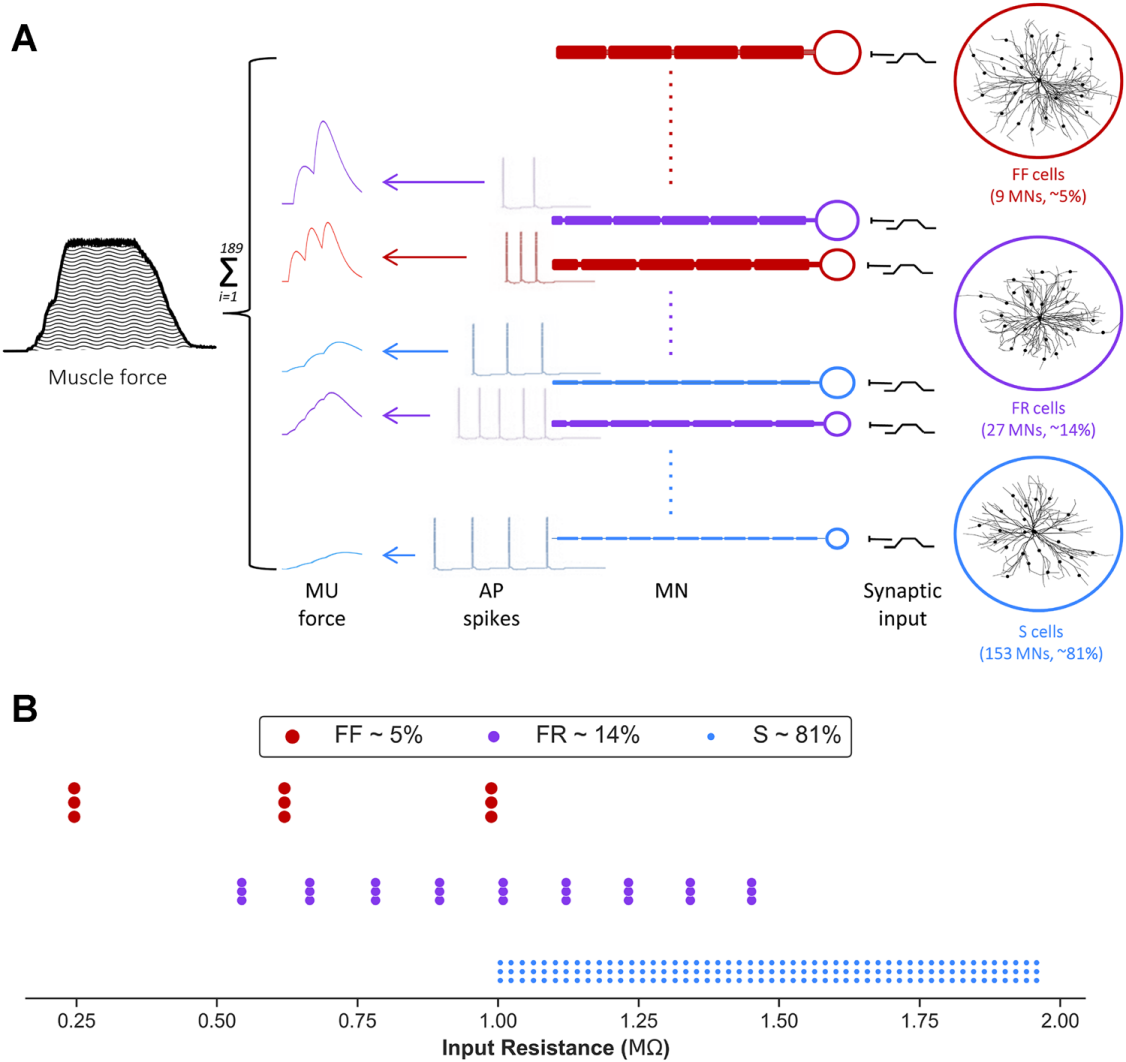
Schematic diagram of the vastus lateralis muscle’s MN pool model structure and cells distribution. **a** Cell morphologies used for the development of S- (blue), FR- (purple), and FF- (red) type MN models. A total of 189 cells were included in the model, with 153 S-type MNs (~ 81%), 27 FR-type MNs (~ 14%), and 9 FF-type MNs (~ 5%). The model was stimulated with synaptic inputs of trapezoidal shape. The spike train of each MN were used to calculate the individual motor unit force; these were summed for the total vastus lateralis muscle force. **b** Distribution of MN type by input resistance within the pool, where each cell type was replicated three times with different spiking threshold to capture the biological variability observed experimentally. *AP * action potential, *FF* fast-fatiguing, *FR *fatgue-resistant, *MN * motoneuron, *MU* motor unit, *S * slow

**Fig. 5:**
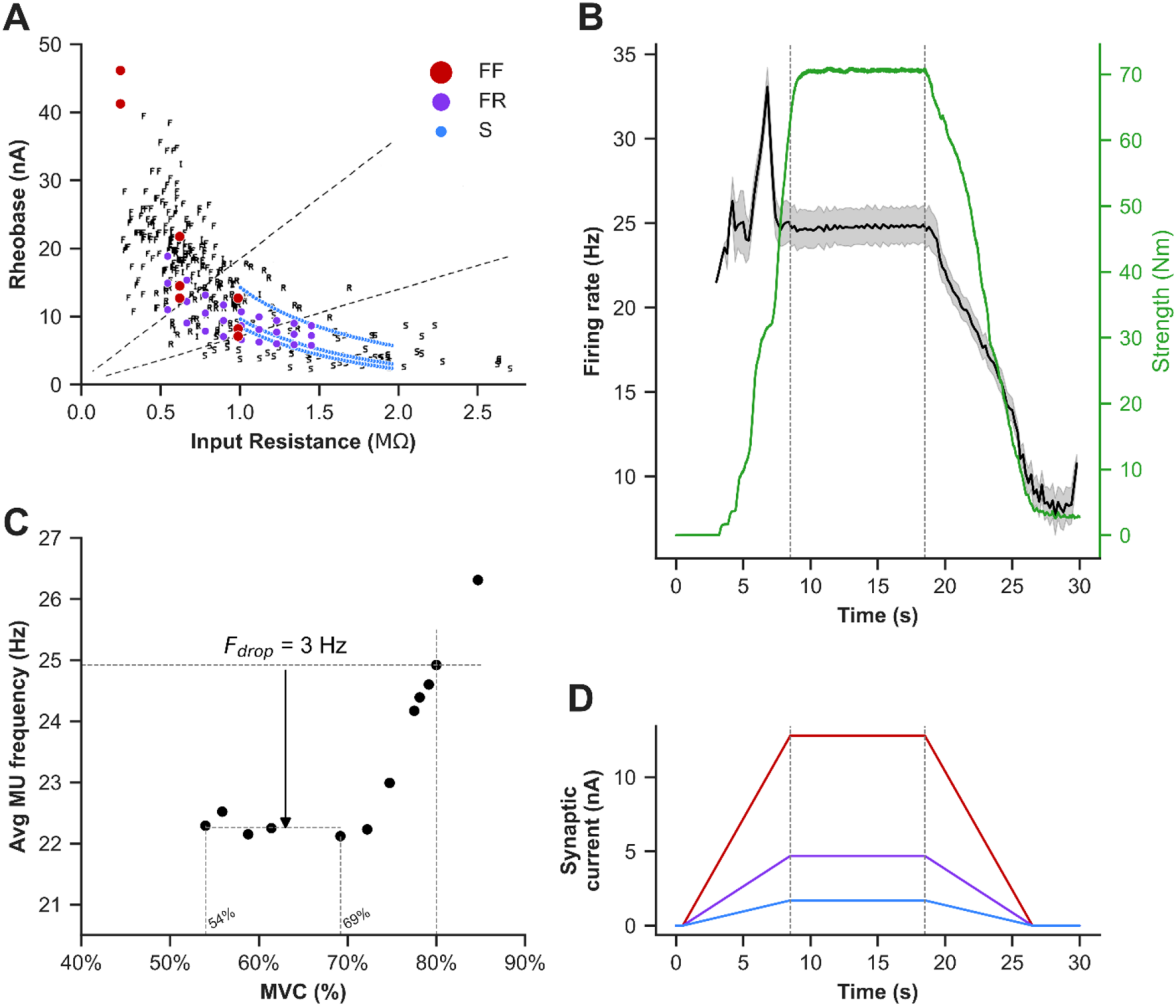
Older adult vastus lateralis muscle MN pool simulations. **a** The rheobase versus input resistance relationship for the pool model (colored dots) overlaid over experimental data from Zengel et al. [52]. **b** MN firing rates (black) with 95% confidence interval (gray) and force (green) during the trapezoidal contraction of 80% MVC (illustrated in [D]). **c** Average motor unit firing rates against the relative voluntary contraction force. Firing rates at 80% MVC is 24.92 Hz. The firing rates were reduced by 3 Hz, while force is reduced to 54–69% of MVC. **d** Effective synaptic input used to stimulate the pool where larger MNs receive higher synaptic currents (FF > FR > S). In panels **a**, **d**: blue, purple, and red refer to S-type MNs, FR-type MNs, and FF-type MNs, respectively. *FF *fast-fatiguing, *FR * fatgue-resistant, *Hz* Hertz, *MN *motoneuron, *MU* motor unit, *MVC* maximal voluntary isometric contraction, *S * slow

Using the vastus lateralis muscle MN pool model, we graded synaptic input to all cell types and calculated the generated vastus lateralis muscle force and mean MUFRs at each submaximal contraction intensity (between 50 and 90% MVC, Figs. 4,  5c). The mean MUFR at 80% MVC in the simulations was 24.92 Hz, which was slightly higher than our in vivo human recordings (see *y*-intercept data in Table 3). When a 3 Hz decrease in MUFR was calculated from the 80% MVC our simulations showed normalized MUFRs that were comparable to those recorded during a torque level between 54 and 69% MVC. In other words, a 3 Hz reduction in mean MUFR resulted in a strength decrement of 11–26%.

As the global reduction of 3 Hz in mean firing rate of the MN pool would cause force reductions due to derecruitment of some cells, as well as the lowered firing rate of other cells, we quantified how much of the reduction in force was a result of each of these two mechanisms. Our simulations show that two-thirds of this force reduction is due to MN derecruitment, whereas one-third is due to the reduced cell firing rate. S-type MNs were derecruited the most (88–95% of the cells, mean of 91%), whereas FR- and FF-type MNs contributed more to force reduction due to the lowering of their firing (47–65% force reduction, mean of 56%).

## Discussion

The purpose of this study was to determine whether older adults with clinically meaningful leg extensor weakness had slower normalized MUFRs relative to older adults without leg extensor weakness via decomposed surface EMG recordings, along with linear regression analysis. Subsequently, we sought to *independently* predict how reductions in normalized MUFRs would affect force output via multi-scale, high-fidelity, anatomically-detailed computational modeling. Lastly, we examined the associations between normalized MUFRs and neuromuscular quality, voluntary activation, and with measures of physical function/mobility in older adults via directionally hypothesized bivariate correlation analysis.

Normalized MUFRs were found to be considerably slower in *weak* older adults relative to *non-weak* older adults at 50% and 80% MVC, whereas in older adults (in general) relative to young adults, considerably slower normalized MUFRs were found only at 80% MVC. While these differences appeared to be more notable in males relative to females, the sex-specific data should be interpreted cautiously due to the small sample size. In addition, significant associations were noted between slower normalized MUFRs and poorer neuromuscular quality, lower voluntary activation, reduced chair rise time, and diminished stair climb power. Interestingly, while females possessed a higher degree of voluntary activation, they generally had lower neuromuscular quality values, chair rise time performance (exception—*non-weak* females), and stair climb power. These novel findings extend prior work [20–23, 32–37], which described differences in MUFR characteristics between older adults (in general) and young adults, to directly investigate to what extent MUFRs are associated with clinically meaningful weakness in older adults. Notably, our findings not only link slowed MUFRs to clinically meaningful leg extensor weakness and impairments in physical function/mobility, but also provide valuable insights on how type-specific MNs and their respective MUs (classified based on anatomical, biophysical, firing, and force properties) contribute to clinically meaningful weakness.

Our findings of slowed normalized MUFRs in older adults (in general) relative to young adults, during moderate-to-high intensity contractions (e.g., ≥ 50% MVC) is largely consistent with the extant literature [20, 21, 23, 33, 34, 37]. Specifically, MUFRs have been reported to be approximately 20–64% lower in older adults (in general) relative to young adults for the intrinsic hand muscles (first dorsal interosseus and abductor digiti minimi) [24, 66] and the lower extremity muscles (tibialis anterior and vastus lateralis) [20, 21, 23, 33, 34, 37]. One notable difference in our work, relative to many previous reports, is that we controlled for the influence of the recruitment threshold on MUFR estimates. Utilizing the y-intercept of the linear regression, we were able to account for the influence of recruitment threshold on each motor unit’s firing behavior. Thus, our observation of 12–14% slower MUFRs during moderate-to-high intensity contractions, which is slightly lower than some prior reports, may be attributed to our normalization procedure. Of note, our normalized MUFR values are similar to those found in prior work that utilized the same muscle and also the y-intercept method [38, 39].

The most crucial and novel finding of our work is the observation that older adults with clinically meaningful leg extensor weakness have significantly slower normalized MUFRs relative to older adults without leg extensor weakness, and that MUFRs are associated with indices of physical function and voluntary (neural) activation. These findings are consistent with prior studies assaying various amplitude characteristics of the interference EMG signal to estimate neural activation, which reported associations with reduced indices of mobility/physical function in older adults [40–46]. Thus, it is plausible that slower MUFRs underlies the findings from these prior studies. In fact, our computational model *independently* predicted that slower MUFRs result in notable strength reductions of 11–26%, with one-third being lost to a reduction in MNFR and two-thirds being lost to MN derecruitment. In addition, slower normalized MUFRs were associated with not only poorer neuromuscular quality, but also with lower levels of voluntary activation and physical function. While the sex-specific findings are likely unpowered, it is interesting to note that the link between MUFR and indices of muscle/physical function appear strongest in men. However, the correlation with voluntary (neural) activation is stronger in women. Together, these novel findings provide evidence for impairments in MUFR being pathogenically linked to age-related weakness, which we postulate is potentially due to dysfunction of MN intrinsic excitability mechanisms.

From a broader perspective, we have previously assessed corticospinal excitability non-invasively using transcranial magnetic stimulation across older adult weakness phenotypes, wherein we reported that *weak* older adults exhibited lower indices of corticospinal excitability [47, 48]. While our prior work did not parse out cortical vs. MN excitability, others have suggested that older adults have lower indices of motor unit excitability relative to young adults. For instance, Christie and Kamen [18] reported that older adults had ~ 10% longer motor unit after-hyperpolarization durations, and very recently, Orssatto et al. [49] noted that older adults had a lower ‘delta frequency’, which is a variable thought to be reflective of a MN’s intrinsic excitability that is heavily influenced by the MN’s persistent inward current amplitude [50, 51]. Thus, one possible explanation for our noted slowed MUFRs in *weak* older adults is that these participants have reduced MN intrinsic excitability, which could be due to MN deterioration itself and/or an impaired monoaminergic system. However, there are other neurological related reasons for why we observed slowed MUFRs, such as reduced net excitatory drive to MN pool or reductions in afferent feedback. Yet, another potential reason may be due to age-related muscular changes (e.g., leftward shift in force-frequency relationship). Hence, further work is needed to better delineate the precise mechanism(s) responsible for slowed MUFRs in age-related weakness. Regardless, our findings suggest neuro-therapeutic approaches that function to increase MUFRs may have clinical utility for treating age-related weakness, with the most effective strategy likely depending on our depth of understanding the biological basis for slowed MUFR patterns.

There are several limitations of our work that should be noted. First, this study was cross-sectional in nature. Thus, our results are not influenced by time (i.e., within-subject aging), and therefore, do not necessarily suggest cause-and-effect relationships. Second, our study population was community-dwelling older adults. Therefore, it is plausible that their MUFR data may not be generalizable to institutionalized older adults. Third, we normalized strength data to thigh lean mass assessed via DXA for muscle quality assessment. Hence, this should be interpreted cautiously as this measure reflects not only the quadriceps muscle group, but also those of other muscles in the thigh region (e.g., adductors, biceps femoris, sartorius), as well as other tissue components (e.g., connective tissue). Fourth, the MUFR work was only recorded from the vastus lateralis muscle, which only accounts for a partial amount the total force generation from the much larger quadriceps femoris muscle group. Thus, it is difficult to know if the other synergistic muscles involved in leg extension force displayed similar MUFR patterns. Fifth, some of our comparisons, particularly those where data were disaggregated by biological sex, were likely underpowered (i.e., *weak* older adults and with voluntary activation measures). Therefore, the multiple comparisons from these data should also be interpreted cautiously. Lastly, we would be remiss if we did not acknowledge that there is robust debate and legitimate concern over the physiological validity/accuracy of surface EMG-derived motor unit recordings. While this approach has advantages (e.g., non-invasive; hence, more feasible in certain populations, recording of higher threshold motor units, increased motor unit yield), the strategy used to evaluate the waveforms identified by a decomposition algorithm can limit the quality of the information and data must be interpreted within this context.

In conclusion, we presented evidence that *weak* older adults have significantly slower normalized MUFRs relative to *non-weak* older adults at moderate-to-high contraction intensities, and that this reduction in MUFR results in a significant strength decrement. Furthermore, we provided valuable insights on how MN types contribute differentially to clinically meaningful force loss. Additionally, we noted significant associations between slower normalized MUFRs and poorer neuromuscular quality, lower voluntary activation, reduced chair rise time, and diminished stair climb power. Taken together, these findings provide evidence that impairments in MUFR are mechanistically linked to clinically meaningful weakness, which provides rational support for the development of neurotherapeutic approaches that function to increase MUFRs to treat age-related weakness.

### Supplementary Information

Below is the link to the electronic supplementary material.

Supplementary file (DOCX 72 kb)

## References

[1] Seeman T, Merkin S, Crimmins E, Karlamangla A (2010). Disability trends among older americans: national health and nutrition examination surveys, 1988–1994 and 1999–2004. Am J Public Health.

[2] Louie G, Ward M (2010). Sex disparities in self-reported physical functioning: true differences, reporting bias, or incomplete adjustment for confounding?. J Am Geriatr Soc.

[3] Cruz-Jentoft A, Bahat G, Bauer J, Boirie Y, Bruyère O, Cooper C (2019). Sarcopenia: revised european consensus on definition and diagnosis. Age Aging.

[4] Manini T, Visser M, Won-Park S, Patel K, Strotmeyer E, Chen H (2007). Knee extension strength cutpoints for maintaining mobility. J Am Geriatr Soc.

[5] Newman A, Kupelian V, Visser M, Simonsick E, Goodpaster B, Kritchevsky S (2006). Strength, but not muscle mass, is associated with mortality in the health, aging and body composition study cohort. J Gerontol A Biol Sci Med Sci.

[6] Visser M, Goodpaster B, Kritchevsky S, Newman A, Nevitt M, Rubin S (2005). Muscle mass, muscle strength, and muscle fat infiltration as predictors of incident mobility limitations in well-functioning older persons. J Gerontol A.

[7] McGrath R, Erlandson K, Vincent B, Hackney K, Herrmann S, Clark B (2019). Decreased handgrip strength is associated with impairments in each autonomous living task for aging adults in the United States. J Frailty Aging.

[8] Rantanen T (2003). Muscle strength, disaiblity and mortality. Scand J Med Sci Sports.

[9] Rantanen T, Avlund K, Suominen H, Schroll M, Frändin K, Pertti E (2002). Muscle strength as a predictor of onset of ADL dependence in people aged 75 years. Aging Clin Exp Res.

[10] Hoffman C, Rice D, Sung H-Y (1996). Persons with chronic conditions. Their prevalence and costs JAMA.

[11] Narici M, Maffulli N (2010). Sarcopenia: characteristics, mechanisms and functional significance. Br Med Bull.

[12] Clark B, Manini T (2008). Sarcopenia ≠ dynapenia. J Gerontol A.

[13] Russ D, Gregg-Cornell K, Conaway M, Clark B (2012). Evolving concepts on the age-related changes in "muscle quality". J Cachexia Sarcopenia Muscle.

[14] Enoka R, Christou E, Hunter S, Kornatz K, Semmler J, Taylor A (2003). Mechanisms that contribute to differences in motor performance between young and old adults. J Electromyogr Kinesiol.

[15] Tieland M, Trouwborst I, Clark B (2018). Skeletal muscle performance and ageing. J Cachexia Sarcopenia Muscle.

[16] Christie A, Kamen G (2006). Doublet discharges in motoneurons of young and older adults. J Neurophysiol.

[17] Christie A, Kamen G (2008). Motor unit firing behavior during prolonged 50% MVC dorsiflexion contractions in young and older adults. J Electromyogr Kinesiol.

[18] Christie A, Kamen G (2010). Short-term training adaptations in maximal motor unit firing rates and afterhyperpolarization duration. Muscle Nerve.

[19] Kamen G, De Luca C (1989). Unusual motor unit firing behavior in older adults. Brain Res.

[20] Kamen G, Sison S, Du C, Patten C (1995). Motor unit discharge behavior in older adults during maximal-effort contractions. J Appl Physiol.

[21] Kamen G, Knight C (2004). Training-related adaptations in motor unit discharge rate in young and older adults. J Gerontol A.

[22] Piasecki M, Ireland A, Stashuk D, Hamilton-Wright A, Jones D, McPhee J (2016). Age-related neuromuscular changes affecting human vastus lateralis. J Physiol.

[23] Connelly D, Rice C, Roos M, Vandervoort A (1999). Motor unit firing rates and contractile properties in tibialis anterior of young and old men. J Appl Physiol.

[24] Kirk E, Gilmore K, Rice C (2018). Neuromuscular changes of the aged human hamstrings. J Neurophysiol.

[25] Kirk E, Gilmore K, Stashuk D, Doherty T, Rice C (2019). Human motor unit characteristics of the superior trapezius muscle with age-related comparisons. J Neurophysiol.

[26] Roos M, Rice C, Connelly D, Vandervoort A (1999). Quadriceps muscle strength, contractile properties, and motor unit firing rates in young and old men. Muscle Nerve.

[27] Dalton B, Harwood B, Davidson A, Rice C (2009). Triceps surae contractile properties and firing rates in soleus of young and old men. J Appl Physiol.

[28] Kallio J, Avela J, Moritani T, Kanervo M, Selänne H, Komi P (2010). Effects of aging on motor unit activation patterns and reflex sensitivity in dynamic movements. J Electromyogr Kinesiol.

[29] Power G, Dalton B, Behm D, Doherty T, Vandervoort A, Rice C (2012). Motor unit survival in lifelong runners is muscle dependent. Med Sci Sport Exerc.

[30] Power G, Dalton B, Behm D, Vandervoort A, Doherty T, Rice C (2010). Motor unit number estimates in masters runners: use it or lose it?. Med Sci Sport Exerc.

[31] Kirk E, Copithorne D, Dalton B, Rice C (2016). Motor unit firing rates of the gastrocnemii during maximal and sub-maximal isometric contractions in young and old men. Neuroscience.

[32] Watanabe K, Holobar A, Kouzaki M, Ogawa M, Akima H, Moritani T (2016). Age related changes in motor unit firing rate pattern of vastus lateralis muscle during low-moderate contraction. Age (Dordr).

[33] Patten C, Kamen G, Rowland D (2001). Adaptations in maximal motor unit discharge rate to strength training in young and older adults. Muscle Nerve.

[34] Patten C, Kamen G (2000). Adaptations in motor unit discharge activity with force control training in young and older human adults. Eur J Appl Physiol.

[35] Dalton B, Jakobi J, Allman B, Rice C (2010). Differential age-related changes in motor unit properties between elbow flexors and extensors. Acta Physiol.

[36] Klass M, Baudry S, Duchateau J (2007). Voluntary activation during maximal contraction with advancing aging: a brief review. Eur J Appl Physio.

[37] Erim Z, Beg M, Burke D, De Luca C (1999). Effects of aging on motor-unit control properties. J Neurophysiol.

[38] De Luca C, Hostage E (2010). Relationship between firing rate and recruitment threshold of motoneurons in voluntary isometric contractions. J Neurophysiol.

[39] Girts R, Mota J, Harmon K, MacLennan R, Stock M (2020). Vastus lateralis motor unit recruitment thresholds are compressed towards lower forces in older men. J Frailty Aging.

[40] Brach J, Kriska A, Newman A, VanSwearingen J (2001). New approach of measuring muscle impairment during a functional task: quadriceps muscle activity recorded during chair stand. J Gerontol A.

[41] Schmitz A, Silder A, Heiderscheit B, Mohoney J, Thelen D (2009). Differences in lower-extremity muscular activation during walking between healthy older and young adults. J Electromyogr Kinesiol.

[42] Clark D, Patten C, Reid K, Carabello R, Phillips E, Fielding R (2010). Muscle performance and physical function are associated with voluntary rate of neuromuscular activation in older adults. J Gerontol A.

[43] Clark D, Manini T, Fielding R, Patten C (2013). Neuromuscular determinants of maximum walking speed in well-functioning older adults. Exp Gerontol.

[44] Clark D, Pojednic R, Reid K, Patten C, Pasha E, Phillips E (2013). Longitudinal decline of neuromuscular activation and power in healthy older adults. J Gerontol A.

[45] Clark D, Reid K, Patten C, Phillips E, Ring S, Wu S (2014). Does quadriceps neuromuscular activation capability explain walking speed in older men and women?. Exp Gerontol.

[46] Reid K, Pasha E, Doros G, Clark D, Patten C, Phillips E (2014). Longitudinal decline of lower extremity muscle power in healthy and mobility-limited older adults: influence of muscle mass, strength, composition, neuromuscular activation and single fiber contractile properties. Eur J Appl Physiol.

[47] Clark L, Manini T, Wages N, Simon J, Russ D (2021). Reduced neural excitability and activation contribute to clinically-meaningful weakness in older adults. J Gerontol A.

[48] Clark B, Taylor J, Hong S, Law T, Russ D (2015). Weaker seniors exhibit motor cortex hypoexcitability and impairments in voluntary activation. J Gerontol A.

[49] Orssatto L, Borg D, Blazevich A, Sakugawa R, Shield A, Trajano G (2021). Intrinsic motoneuron excitability is reduced in soleus and tibialis anterior of older adults. Geroscience.

[50] Gorassini M, Yang J, Siu M, Bennett D (2002). Intrinsic activation of human motoneurons: possible contribution to motor unit excitation. J Neurophysiol.

[51] Heckman C, Gorassini M, Bennett D (2005). Persistent inward currents in motoneuron dendrites: implications for motor output. Muscle Nerve.

[52] Zengel J, Reid S, Sypert G, Munson J (1985). Membrane electrical properties and prediction of motor-unit type of medial gastrocnemius motoneurons in the cat. J Neurophysiol.

