# Supplementary material for: Reductions in Motor Unit Firing are Associated with Clinically Meaningful Leg Extensor Weakness in Older Adults
Source: Calcif Tissue Int. 2023 Aug 21;114(1):9–23. doi: 10.1007/s00223-023-01123-x (PMC10791983; doi:10.1007/s00223-023-01123-x)
Supplement: Supplementary file 1 — Supplementary file (DOCX 72 kb) [file 223_2023_1123_MOESM1_ESM.docx]

**SUPPLEMENTAL MATERIAL FOR:**

**REDUCTIONS IN MOTOR UNIT FIRING ARE ASSOCIATED WITH CLINICALLY MEANINGFUL**

**LEG EXTENSOR WEAKNESS IN OLDER ADULTS**

Nathan P. Wages, Ph.D.**^1, 2,3^**, Mohamed H. Mousa, M.S.**^4^**, Leatha A. Clark, DPT, M.S.**^1, 2, 5^**, Dallin Tavoian, Ph.D.**^1, 6^**, W. David Arnold, M.D.**^7^**, Sherif M. Elbasiouny, Ph.D.**^3, 4^**, and Brian C. Clark, Ph.D.**^1, 2, 8^**

**^1^** Ohio Musculoskeletal and Neurological Institute, Ohio University, Athens, OH, USA

**^2^** Department of Biomedical Sciences, Ohio University, Athens, OH, USA

**^3^** Department of Neuroscience, Cell Biology & Physiology, Wright State University, Dayton, OH, USA

**^4^** Department of Biomedical, Industrial & Human Factors Engineering, Wright State University, Dayton, OH, USA

**^5^** Department of Family Medicine, Ohio University, Athens, OH, USA

**^6^** Department of Physiology, University of Arizona, Tucson, AZ, USA

**^7^** NextGen Precision Health, The University of Missouri, Columbia, MO, USA

^8^ Division of Geriatric Medicine, Ohio University, Athens, OH, USA

**Journal: *Calcified Tissue International and Musculoskeletal Research***

**Corresponding authors:**

Nathan P. Wages

353 Irvine Hall

1 Ohio University

Athens, OH, 45701

wages@ohio.edu

Brian C. Clark

250 Irvine Hall

1 Ohio University

Athens, OH, 45701

clarkb2@ohio.edu

Sherif M. Elbasiouny

350 NEC Building

3640 Colonel Glenn Highway

Dayton, OH, 45435

sherif.elbasiouny@wright.edu

**SUPPLEMENTAL METHODS**

**General Overview.** Forty-three older adults and 24 young adults participated in this study. Participants had their non-dominant isokinetic and isometric leg extensor strength, as well as their handgrip strength, assessed. Participants also performed physical function/mobility tests, body composition assessments *via* dual-energy x-ray absorptiometry (DXA), and completed trapezoidal, target torque matching tasks at contraction intensities of 20%, 50%, and 80% of their maximal volitional contraction (MVC). Decomposed surface electromyographic (EMG) recordings were used to estimate MUFRs from the non-dominant vastus lateralis muscle during these target torque matching tasks.

The primary outcome of interest was the y-intercept calculated from the mean MUFR versus the recruitment threshold scatterplot fit with a linear regression (calculated on a subject-by-subject basis at each contraction intensity; see Figure 1). The y-intercept method, as opposed to using simply MUFRs at given contraction intensities, was preferred because it acts as a normalization process adjusting for the inherent influence of each motor unit’s recruitment threshold on its respective firing rate [1, 2] (see section ‘*Determination of Normalized Mean Motor Unit Firing Rates [MUFRs]’* for further details). For clarity, henceforth the y-intercept data will be referred to as ‘normalized MUFR’. Subsequently, a multi-scale, high-fidelity, anatomically-detailed computational model was used to *independently* predict how reductions in normalized MUFR would negatively impact strength in older adults. Additionally, associations between normalized MUFR and indices of lean mass, voluntary activation, and physical function/mobility were performed *via* bivariate correlations.

**Participants:** Forty-three community-dwelling older adults (63-90 years, mean: 75.4±7.4 years; 46.5% female) and 24 young adults (19-25 years, mean: 22.0±1.8 years; 58.3% female) were included in the primary analysis (see Tables 1 and 2). Participants were between either 18-25 years or ≥60 years of age and had a body mass index between 18-40 kg/m^2^. Participants were living independently and were free of overt musculoskeletal and neurological disease. Participants were instructed to abstain from consuming caffeine for >4 hours and alcohol for >24 hours prior to the testing session. The Ohio University Institutional Review Board approved this study, and all study participants provided written informed consent in accordance with the Declaration of Helsinki.

To characterize the older adults, we measured their 1) six-minute walk gait speed (on a 30-meter walkway with a left hand turn around a cone), 2) short physical performance battery (SPPB) test (composite score based on balance, chair rise time, and short-distance gait speed), 3) stair climb speed, and 4) comorbidities *via* the Charlson Comorbidity Index. These tests have been described previously [3, 4]. In brief, the six-min walk gait speed test entails participants walking as quick and as far as possible in 6 min (on a 30-m course). Specifically, participants are instructed to walk in a straight line for 30 m, complete a 180° turn to the left around a cone and walk back to the starting line, which was an additional 30 m. Once participants arrived at the starting line, they completed another 180° turn to the left around an additional cone and proceeded to repeat this sequence as many times as possible. Distance is then divided by time to determine gait speed. The SPPB test entails: 1) static balance tests (feet are placed side-by-side, semi-tandem, and in tandem), 2) a 5x chair rise test (participants transition between sitting-to-standing-to-sitting again as quick as possible 5 times), and 3) 4-meter walk (walking at one’s normal pace). The scores from the 3 events are summed together with a maximum score of 12. The stair climb test entails participants climbing a flight of stairs (8 steps) as quickly as possible. They are instructed not to use the handrail unless they are unable to climb the stairs without its use. The time required to complete the test was measured using switch mats (Lafayette Instruments Model 63516A) interfaced with a digital timer (Lafayette Instruments Model 54060). Stair climb power is then calculated via the power equation: power (Watts) = body weight (kgs) x 9.8 m/s^2^ x (stair height [meters] divided by stair climb time [seconds]). And finally, the Charlson Comorbidity Index entails categorizing comorbidities with associated weights ranging from 1-6 to determine predicted risk of mortality over a certain period of time. In all participants, we assessed body composition (including appendicular and thigh lean mass) *via* DXA (Hologic Discovery QDR model Series, Waltham, MA, USA), time of moderate-to-vigorous intensity physical activity *via* accelerometry (ActiGraph, wgt3x-bt, Pensacola, FL), and neuropsychological status *via* the Repeatable Battery for the Assessment of Neuropsychological Status (RBANS). See Tables 1 and 2.

**Leg Extension and Handgrip Strength:** Non-dominant, isometric and isokinetic leg extension strength measures were recorded *via* a dynamometer (Biodex System 4 Dynamometer, Biodex Medical Systems Inc., Shirley, NY) as previously described [4]. Older adult clinically meaningful weakness phenotypes were determined using the same isokinetic leg extension strength protocol that was performed in the Health, Aging, and Body Composition Study from which the leg extensor weakness thresholds were derived [5] (see below for further details). Participants performed three MVCs for the isometric trials with one min of rest between each bout and six MVC for the isokinetic trials with 30 sec of rest between bouts. We selected to test the non-dominant leg as radiographic evidence suggests higher incidences of knee osteoarthritis for the dominant leg in older adults [6]. Note that previous work has shown isometric and isokinetic testing to have high reliability (intraclass correlation coefficients (ICCs) >0.90) [7, 8].

Maximal handgrip strength was assessed in a manner similar to that described by Villanfañe, et al., [9] using a portable JAMAR® Hydraulic Hand Dynamometer; (Model 5030 J1; Lafayette Instrument Co.; Lafayette, Indiana). Three MVC were performed with 30 sec of rest between bouts and measurements were recorded to the nearest 1.0 kg. If the MVCs were not within 3 kgs an additional MVC was performed. The average of the highest three MVCs were recorded for each hand.

**Clinically Meaningful Weakness Classifications:** As stated above, older adult clinically meaningful weakness phenotypes were determined using the same isokinetic leg extension strength protocol that was performed in the Health, Aging, and Body Composition Study from which the leg extensor weakness thresholds were derived [5]. Specifically, older adults were classified as ‘*weak*’ for the leg extensor muscles if their non-dominant isokinetic (60°/sec) strength relative to their body weight was ≤1.12 Nm/kg for men and ≤1.00 Nm/kg for women. This weakness classification represents the 1st & 3rd decile (for men & women, respectively) of sex-specific, relative strength values, which has been shown to be predictive of non-disabled older adults subsequently developing severe mobility limitations [5]. These cut points were derived from a cohort of 1,355 men and 1,429 women (mean: 73.6±2.85 years) that were tracked over ~5.9 years [5]. Older adults with values above these sex-specific cut points were classified as ‘*non-weak*’. Of note, all young adults were classified as ‘*non-weak*’ as their values were above the sex-specific 1st & 3rd decile cut points. All assessments conducted were performed by lab personnel blinded to weakness classification.

**Neuromuscular Quality**: Isokinetic leg extensor strength of the non-dominant leg was expressed relative to thigh lean mass, obtained *via* whole body DXA scans as previously described (Hologic Discovery QDR model Series, Waltham, MA, USA) [10, 11]. Calculation of whole-body, appendicular, and non-dominant thigh lean tissue mass were performed using the analysis package (Hologic APEX, ver. 4.0.2). Care was taken to follow The International Society for Clinical Densitometry guidelines for positioning during the scan [12]. While DXA-derived lean mass has received notable criticisms in recent years about its insensitivity to detect small differences or changes over time [10], it does appear to yield a reasonable estimate of muscle mass in cross-section studies, in that, DXA-derived lean mass is highly correlated with MRI-derived skeletal muscle mass for whole body (r = 0.94; P < 0.001) and leg region (r = 0.91; P < 0.001) [13].

**Voluntary Activation**: Here, the doublet interpolation technique was performed similar to our previous description [14]. In short, large (e.g., 3 × 4 or 4 × 5-inch electrodes depending on the quadriceps size) self-adhesive electrodes were applied over the motor points of the non-dominant rectus femoris and vastus medialis muscles. While participants were resting with their knee positioned at 90°, single pulses of electrical stimuli (200 microsec duration) were applied at gradual increases in current, with a constant voltage (400 V), until a plateau was reached in the evoked force output (DS7AH; Digitimer, Hertfordshire, United Kingdom). Next, participants performed one-to-two 5-second isometric MVCs with a 100-Hz supramaximal doublet being delivered during the peak force output followed by a second doublet delivered to the resting muscle. The increase in force immediately following the stimulation was expressed relative to a potentiated response evoked by the same doublet applied at rest (i.e., one-to-two seconds after the supramaximal doublet stimulation during the MVC). We have previously reported that the assessment of voluntary activation in the lower extremity musculature is repeatable with the plantar flexors exhibiting a ~3% coefficient of variation across days [7, 8]. We should also mention that 10 older adults and two young adults were unable to complete the voluntary activation testing due to discomfort associated with the stimulation.

**Motor Unit Recordings and Analyses**: Trapezoidal, isometric contractions were performed using the same mechanical setup as described for the leg extension strength measures at 20%, 50%, and 80% MVC. Target torque matching templates contained, in the following order a(n), 1) quiescent period (4 sec), 2) ascending phase (10% MVC/sec), 3) steady-state, plateau phase (10 sec for the 20% and 50% MVC, and 8 sec for the 80% MVC), 4) descending phase (10% MVC/sec), and 5) additional quiescent period (4 sec). All torque matching templates were completed twice with 1 min of rest between bouts.

Surface EMG signals were recorded from the non-dominant vastus lateralis muscle *via* a Bagnoli Desktop system (Delsys, Inc., Natick, MA). Prior to testing, the skin over the vastus lateralis muscle and the patella of the alternate leg were shaved, cleansed with an alcohol pad, and abraded with sandpaper. The sensor was placed in accordance with recommendations by Zaheer et al. (2012) [15]. Signals were detected with a 5-pin array EMG sensor (Delsys, Inc., Natick, MA). These signals were differentially amplified, filtered with a bandwidth of 20 Hz to 450 Hz, and sampled at 20 kHz. Signal quality (i.e., signal-to-noise ratio >3.0, baseline noise value <2.0 uV root-mean-square, line interference <1.0) was verified for a 20% contractile intensity prior to data acquisition. The filtered EMG signals served as the input to the Precision Decomposition III algorithm, which was utilized *via* dEMG Analysis software (ver. 1.1, Delsys, Inc., Natick, MA). Decomposition-Synthesize-Decomposition-Compare testing was used to remove motor units with detection accuracy <90.0% [16]. Before analysis, all MUFR curves were smoothed by low-pass filtering each motor unit’s impulse train with a 1-sec Hanning window.

*Selection Criteria for Analyzing ‘Best’ Torque Matching Trial*: For analyses, a single trial was selected at each respective contraction intensity. Our selection process was as follows. First, a trial had to have at least six motor units to be considered valid as it has been suggested that an EMG recording that decomposed a minimum of five MUs can accurately represent the MUFR behavior for the entire motor unit pool [17, 18]. Second, one investigator (NPW) visually inspected the torque tracing(s) to ensure that the trial was performed accurately (e.g., no drastic over- or under-shooting the force trajectory line). If multiple trials had an acceptable number of motor units and were deemed to have acceptable torque matching accuracy, the trial with the widest motor unit distribution across the recruitment threshold range was selected for analysis. Additionally, during the visual inspection process, motor units that were identified as being recruited during the quiescent period, or during the steady state plateau phase, were excluded from analysis.

Of note, in our full dataset we obtained motor unit recordings from 65 older adults and 26 young adults. However, due to the quality control and assurance criteria listed above, 22 older adults and two young adults were excluded from further analysis. The primary reason for exclusion was contraction intensity compliance (i.e., there were no acceptable trials for any of the three different contraction intensities). Comparative details (aggregated and disaggregated by sex) between older adults used for further analysis versus those removed from analysis are provided in the Supplementary Tables 1 and 2.

*Determination of Normalized Mean Motor Unit Firing Rates (MUFRs)*: Current methods to assay mean MUFRs for different populations vary from collapsing MUFRs across participants at different contraction intensities or across all contraction intensities [19-23] to analyzing MUFRs on a participant-by-participant basis at each contraction intensity before averaging the MUFRs data [24-26]. Moreover, there are also a variety of methods to interpret mean MUFRs data, such as organizing MUs by ‘recruitment or frequency bins’ [27-29], or *via* a coefficient of the linear regression for the mean MUFRs versus the recruitment threshold scatterplot [30-32] (i.e., y-intercept and/or slope). While each method has its advantages, the potential differences in results may influence one’s interpretation and possible clinical significance.

For this study, the mean MUFR data was individually normalized at each contraction intensity. Here, the y-intercept from the mean MUFR versus recruitment threshold scatterplot fit with a linear regression was used as this approach theoretically controls for the ‘onion skin phenomenon’ of MUFRs (i.e., larger-threshold motor units fire slower than lower-threshold motor units) [33]. Specifically, for each participant at each contraction intensity (20%, 50%, and 80% MVC) the coefficients of the linear regression (y-intercept and slope) were calculated for the mean MUFRs versus the recruitment threshold scatterplot. Thus, the y-intercepts (our index for ‘*normalized MUFR*’) and slopes were determined for each participant in a particular group, at each contraction intensity, before averaging the aggregated data by group and subsequently disaggregating the data by sex. The coefficients of the linear regression for mean MUFR versus recruitment threshold of the vastus lateralis muscle has been reported to have high intra- and inter-day reliability (determined *via* ICCs) ranging from 0.766-0.824 and 0.867-0.919 for the slopes and from 0.780-0.915 and 0.804-0.927 for the y-intercepts, respectively [34].

**Computational Modeling**: Developed with NEURON simulation environment (ver. 7.6). Simulations were run on the Neuroscience Gateway [Stampede2 KNL](https://nsgprod.sdsc.edu:8443/portal2/createTask!selectTool.action?selectedTool=NEURON74_STAMPEDE2KNL) Super-computer with NEURON 7.6 tool. Analysis was performed in *MATLAB* (ver. 9.9.0 [R2020b]). For the vastus lateralis computer model employed herein, this model was based on the work of Allen and Elbasiouny (2018) [35] in which electrical properties of model MNs were rigorously examined against multiple sets of experimental data (using repeated, randomized statistical analyses) and the model parameters have undergone sensitivity analysis to confirm the validity of the simulation results. Additionally, as the vastus lateralis MN model incorporated several features of biological variability (i.e., heterogeneity and overlap in cell properties of different MN types), this model simulates MN recruitment, firing rates, and force generation more accurately than computer models missing those features. *See below for futher details.*

*Vastus lateralis motoneuron pool model – individual cells:* Cat MNs, as opposed to rodents, were used as the foundation of our human cell models as their electrical and firing properties, as well as force production, are very similar to humans [36, 37]. Specifically, the reconstructed morphologies of identified slow (S), fatigue-resistant (FR), and fast-fatiguing (FF) cat cells were used to represent the three-dimensional anatomy of model MNs (Figure 4A). To mimic the physiological activation of MNs *via* synaptic inputs, excitatory synapses were distributed over the dendrites of each model cell following the realistic distribution of Ia afferent-to-motoneuron contacts labeled intracellularly with horseradish peroxidase in type-identified cat MNs [38-40]. Additionally, the model MNs include somatic and dendritic voltage-gated and calcium-activated ion channels that mediate transient and persistent inward/outward currents underlying nonlinear MN firing bistability [41-43]. Moreover, their membrane biophysical (i.e., mathematical formulizations to represent the physical properties of the MN) and electrical properties matched those measured experimentally from cat MN [44] (see Figure 5A).

*Vastus lateralis motoneuron pool model – synaptic input:* Individual cells in the MN pool model were stimulated through synaptic inputs with trapezoidal activation waveforms, like in our human experimental protocols. The conductance of these synaptic inputs was determined using effective synaptic currents of pyramidal inputs to spinal MNs, in which large MNs received higher synaptic current than small MNs (Figure 5D; also see Figure 4E in Powers & Binder [2001] [45]). The mean MUFR of recruited motor units was measured in the simulation *via* a 200 ms moving window (during the plateau phase of trapezoidal waveforms).

*Vastus lateralis motoneuron pool model – motor unit type composition*: Using methodology from Allen & Elbasiouny (2018) [35], the percentage of different cell types (S, FR, FF) in the vastus lateralis muscle MN pool was determined using older adult fiber type data from the Staron et al., (2000) [46] and Marzani et al., (2005) [47] articles. Fifty-eight percent of motor units were identified as Type I (S), 25% were Type IIA (FR), and 17% were Type IIB (FF). Given that information on innervation ratios of the human vastus lateralis muscle is lacking in the literature, we found information from cats [48, 49] and human modeling studies [50, 51] to estimate the average innervation ratio: 1 (Type I): 2.5 (Type IIA): 5 (Type IIB). Thus, the innervation ratio for Type IIA is 2.5 times greater than the innervation ratio for Type I, while the innervation ratio for Type IIB is 5 times greater than the innervation ratio with Type I. Based on these muscle fiber types and innervation ratio data, the MN pool innervating the vastus lateralis muscle for older adults consisted of ~81% S-type, ~14% FR-type, and ~5% FF-type cells. As the vastus lateralis muscle MN pool was modeled with 189 model cells in the present study, 153 were S-type, 27 were FR-type, and 9 were FF-type cells (Figure 4A).

*Vastus lateralis motoneuron pool model – force generation*: Here, spike trains were converted to twitch forces in each cell in the MN pool model, and all motor unit forces were summated to simulate the total vastus lateralis muscle force (Figure 5A). As force data from type-identified motor units are not available in humans, the force output of each motor unit in the model was based on the contractile properties of young adult cats from Burke et al. (1971) [52] measured from type-identified motor units (S, FR, FF). To simulate the changes in force production with aging, we adjusted the motor unit force parameters (twitch force amplitude and duration) by the percent changes observed from young adults to older adults (i.e., twitch force amplitude was decreased by 36% and twitch force duration was increased by 15%). To simulate the human force measurements, the total force generated from the cat vastus lateralis muscle MN pool model was scaled up to match the older adult data. Then, the synaptic drive to the MNs was reduced to record the change in predicted strength (Figure, 5B, secondary y-axis).

**Statistical Analysis:** Repeated measures analysis of covariances (ANCOVAs) were performed to examine group differences in the respective dependent variable (e.g., normalized MUFR). Here, group (e.g., *weak* versus *non-weak* older adults) was a between-participant factor and contraction intensity were a within-participant factor (3 levels). Sidak post-hoc analyses were performed if a significant main effect of interaction was observed. One-way ANCOVAs were used for group wise comparisons. Sex was covaried in all aggregated data analyses. Consistent with NIH recommendations for sex as a biological variable [53]. Additionally, directionally hypothesized bivariate correlations (i.e., one-tailed tests) were used to examine whether there were associations between slower normalized MUFRs and poorer neuromuscular quality, lower voluntary activation, and with reduced physical function/mobility in older adults. A p-value of ≤0.05 was required for statistical significance. Statistical Package for the Social Sciences (SPSS; version 25.0, Chicago, IL) was used for data analysis and presented as estimated marginal means ± the standard error of mean (SEM). Effect sizes (ƞ2) are also reported to aid in interpretation.

**References**

1. De Luca, C.J. and E.C. Hostage, *Relationship between firing rate and recruitment threshold of motoneurons in voluntary isometric contractions.* J Neurophysiol, 2010. **104**(2): p. 1034-46.

2. Contessa P, D.L.C., Kline J, *The compensatory interaction between motor unit firing behavior and muscle force during fatigue.* J Neurophysiol 2016. **116**: p. 1579-1585.

3. Riwniak, C., Simon, J., Wages, N., Clark, L., Manini, T., et al., *Comparison of a multi-component physical function battery to usual walking speed for assessing lower extremity function and mobility limitations in older adults.* J. Nutr. Health Aging, 2020. **24**: p. 906-913.

4. Wages, N., Simon, J., Clark, L., Amano, S., Russ, D., et al., *Relative contribution of muscle strength, lean mass, and lower extremity motor function in explaining between-person variance in mobillity in older adults.* BMC Geriatrics 2020. **20**: p. 255

5. Manini, V., Won-Park, Patel, Strotmeyer, Chen, Goodpaster, Rekeneire, Newman, Simonsick, Kritchevsky, Ryder, Schwartz, Harris, *Knee extension strength cutpoints for maintaining mobility.* Journal of the American Geriatrics Society, 2007. **55**(3): p. 451-457.

6. Neame, R., Zhang, W., Deighton, C., Doherty, M., Doherty, S., et al., *Distribution of radiographic osteoarthritis between the right and left hands, hips, and knees. .* Arthritis Rheum., 2004. **50**: p. 1487-1494.

7. Clark B, C.S., Ploutz-Snyder L, *Reliability of techniques to assess human neuromuscular function in vivo.* J Electromyogr Kinesiol, 2007. **17**: p. 90-101.

8. Sole G, H.J., Milosavljevic S, Nicholson H, Sullivan S, *Test-retest reliability of isokinetic knee extension and flexion.* Arch Phys Med Rehabil, 2007. **88**: p. 626-631.

9. Villafañe, J., Valdes, K., Vanti, C., Pillastrini, P., Borboni, A. , *Reliability of handgrip strength test in elderly subjects with unilateral thumb carpometacarpal osteo-arthritis.* Hand, 2015. **10**: p. 205-209.

10. Tavoian, D., Ampomah, K., Amano, S., Law, T., Clark, B., *Changes in DXA-derived lean mass and MRI-derived cross-sectional area of the thigh are modestly associated.* Sci. Rep., 2019. **9**: p. 10028.

11. Clark, L., Manini, T., Wages, N., Simon, J., Russ, D., et al., *Reduced neural excitability and activation contribute to clinically-meaningful weakness in older adults. .* J. Gerontol. A Biol. Sci. Med. Sci., 2021. **76**: p. 692-702.

12. Hangartner, T., Warner, S., Braillon, P., Jankowski, L., Shepard, J. , *The official positions of the international society for clinical densitometry: acquisition of dual-energy X-ray absorptiometry body composition and considerations regarding analysis and repeatability of measures.* J. Clin. Densitom. , 2013. **16**: p. 520-536.

13. Chen Z, W.Z., Lohman T, Heymsfield S, Outwater E, Nicholas J, et al., , *Dual-energy X-ray absorptiometry is a valid tool for assessing skeletal muscle mass in older women. .* J Nutr, 2007. **137**: p. 2775-2780.

14. Russ, D.W., et al., *Evolving concepts on the age-related changes in "muscle quality".* J Cachexia Sarcopenia Muscle, 2012. **3**(2): p. 95-109.

15. Zaheer, F., S.H. Roy, and C.J. De Luca, *Preferred sensor sites for surface EMG signal decomposition.* Physiol Meas, 2012. **33**(2): p. 195-206.

16. Nawab, S.H., S.S. Chang, and C.J. De Luca, *High-yield decomposition of surface EMG signals.* Clin Neurophysiol, 2010. **121**(10): p. 1602-15.

17. Negro, F. and D. Farina, *Decorrelation of cortical inputs and motoneuron output.* J Neurophysiol, 2011. **106**(5): p. 2688-97.

18. Gallego J, D.J., Holobar A, Ibanez J, Pons J, Louis E, Rocon E, Farina D., *Influence of common synaptic input to motor neurons on the neural drive to muscle in essential tremor.* J Neurophysiol, 2015. **113**: p. 182-191.

19. Erim, Z., et al., *Effects of aging on motor-unit control properties.* J Neurophysiol, 1999. **82**(5): p. 2081-91.

20. Farina, D., et al., *Adjustments differ among low-threshold motor units during intermittent, isometric contractions.* J Neurophysiol, 2009. **101**(1): p. 350-9.

21. Farina, D., et al., *Decoding the neural drive to muscles from the surface electromyogram.* Clin Neurophysiol, 2010. **121**(10): p. 1616-23.

22. Farina, D., et al., *The Extraction of Neural Information from the Surface EMG for the Control of Upper-Limb Prostheses: Emerging Avenues and Challenges.* IEEE Trans Neural Syst Rehabil Eng, 2014.

23. Del Vecchio A, N.F., Felici F, Farina D, *Associations between motor unit action potential parameters and surface EMG features.* J Appl Physiol, 2017. **123**: p. 835– 843.

24. Patten, C., Kamen, G., Rowland, D. , *Adaptations in maximal motor unit discharge rate to strength training in young and older adults. .* Muscle Nerve, 2001. **24**: p. 542-550.

25. Stock M, M.J., *Shifts in the relationship between motor unit recruitment thresholds versus derecruitment thresholds during fatigue.* Med Engineer Phys 2017. **50**: p. 35-42.

26. Stock M, T.B., *Effects of barbell deadlift training on submaximal motor unit firing rates for the vastus lateralis and rectus femoris.* Plos One, 2014. **9**: p. e115567.

27. Mottram C, C.E., Meyer F, Enoka R, *Frequency modulation of motor unit discharge has task-dependent effects on fluctuations in motor output.* J Neurophysiol, 2005. **94**: p. 2878-2887.

28. Defreitas, J.M., et al., *Synchronization of low- and high-threshold motor units.* Muscle Nerve, 2014. **49**(4): p. 575-83.

29. Nishikawa Y, H.A., Watanabe K, Takahashi T, Ueno H, Maeda N, Maruyama H, Tanaka S, Hyngstrom A, *Detecting motor unit abnormalities in amyotrophic lateral sclerosis using high-density surface EMG.* Clin Neurophysiol, 2022. **142**: p. 262-272.

30. Sterczala A, M.J., Trevino M, Dimmick H, Herda T, *Differences in the motor unit firing rates and amplitudes in relation to recruitment thresholds during submaximal contractions of the first dorsal interosseous between chronically resistance-trained and physically active men.* Appl Physiol Nutr Metab, 2018. **43**: p. 759-768.

31. Trevino M, H.T., Fry A, Gallagher P, Vardiman J, Mosier E, Miller J, *Influence of the contractile properteis of muscle on motor unit firing rates during a moderate-intensity contraction in vivo.* J Neurophysiol, 2016. **116**: p. 552-562.

32. Girts, R., Mota J, Harmon K, MacLennan R, Stock M, *Vastus Lateralis Motor Unit Recruitment Thresholds are Compressed Towards Lower Forces in Older Men.* J Frailty Aging, 2020. **9**: p. 191-196.

33. P., D.L.C.C., *Biomechanical benefits of the onion-skin motor unit scheme.* J Biomech, 2015. **48**: p. 195-203.

34. Colquhoun R, T.P., Magrini M, Muddle T, Jenkins N, *The influence of input excitation on the inter- and intra-reliability of the motor unit firing rate versus recruitment threshold relationship.* J Neurophysiol, 2018. **120**: p. 3131-3139.

35. Allen J, E.S., *The effects of model composition design choices on high-fidelity simulations of motoneuron recruitment and firing behaviors. .* Journal of Neural Engineering 2018. **15**(3).

36. Jones, K.B.P., *Computer simulation of the responses of human motoneurons to composite 1a epsps: effects of background firing rate.* J Neurophysiol, 1997. **77**: p. 405-420.

37. Manuel, M.H.C., *Adult mouse motor units develop almost all of their force in the subprimary range: a new all-or-none strategy for force recruitment?* J Neurosci 2011. **31**: p. 15188-15194.

38. Burke, R., Walmsley B, Hodgson J, *HRP anatomy of group Ia afferent contacts on alpha motoneurons.* Brain Res, 1979. **160**: p. 347-352.

39. Glenn, L., Burke, R., Fleshman, J., Lev-Tov, A., *Estimates of electrotonic distance of group Ia contacts on cat a-motoneurons: an HRP-morphological study.* Soc Neurosci Abstr, 1982. **8**: p. 995.

40. Burke, R.G.L., *Horseradish peroxidase study of the spatial and electrotonic distribution of group Ia synapses on type-identified ankle extensor motoneurons in the cat.* J Comp Neural, 1996. **372**: p. 465-485.

41. Elbasiouny S, B.D., Mushahwar V, *Simulation of dendritic Cav1.3 channels in cat lumbar motoneurons: spatial distribution.* J Neurophysiol, 2005. **94**: p. 3961-3974.

42. Elbasiouny S, B.D., Mushahwar V, *Role of persistent inward currents (PICs) in enhancement of integration of synaptic inputs in spinal motoneurons.* Abstract Viewer/Itinerary Planner, Washington, DC: Society for Neuroscience, Online. Prog. No. 750.4, 2005.

43. S, M.M.E., *Dendritic distributions of L-type Ca(2+) and SKL channels in spinal motoneurons: a simulation study.* J Neurophysiol, 2020. **124**: p. 1285-1307.

44. Fleshman, J., Segev, I., Burke, R, *Electrotonic architecture of type-identified alpha-motoneurons in the cat spinal cord.* J Neurophysiol, 1988. **60**: p. 60-85.

45. M, P.R.a.B., *Distribution of oligosynaptic group I input to the cat medial gastrocnemius motoneuron pool.* Journal of Neurophysiology 1985. **53**(2): p. 497-517.

46. Staron, R., Hagerman, F., Hikida, R., Murray, T., Hostler, D., Crill, M., et al., *Fiber type composition of the vastus lateralis muscle of young men and women.* J Histochemistry Cytochemistry, 2000. **48**: p. 623–629.

47. Marzani, B., Felzani, G., Bellomo, R., Vecchiet, J., Marzatico, F., *Human muscle aging: ROS-mediated alterations in rectus abdominis and vastus lateralis muscles.* Experimental Gerontology, 2005. **40**: p. 959–965.

48. Bodine, S., Roy, R., Eldred, E., Edgerton, V., *Maximal force as a function of anatomical features of motor units in the cat tibialis anterior. .* J. Neurophysiol., 1987. **57**: p. 1730–1745.

49. Burke, R., Levine, D., Tsairis, P., Zajac, F., *Physiological types and histochemical profiles in motor units of the cat gastrocnemius.* J. Physiol., 1973. **234**: p. 723–748.

50. Ahad, M., *Analysis of Simulated Electromyography (EMG) Signals Using Integrated Computer Muscle Model. .* <https://trace.tennessee.edu/utk_graddiss>, 2007.

51. Enoka, R., & Fuglevand, A., *Motor unit physiology: Some unresolved issues.* Muscle Nerve, 2001. **24**: p. 4–17.

52. Burke, R., Levine, D., Zajac, F., *Mammalian motor units: physiological-histochemical correlation in three types in cat gastrocnemius.* Science 1971. **174**(709-712).

53. NIH, *Consideration of Sex as a Biological Variable in NIH-funded Research.* 2015.

**Supplementary Table 1 (Aggregated). Descriptive Characteristics for Older Adults Possessing Acceptable and Unacceptable Trials for Motor Unit Recordings (mean ± SD).**

| *Older Adults* | | |  |
| --- | --- | --- | --- |
|  | **With Acceptable**  **Motor Unit Recordings**  **N=43** | | **With Unacceptable**  **Motor Unit Recordings**  **N=22** |
| Age (years) | 75.4±7.4 | | 74.5±5.7 |
| Women (%) | 46.5 | | 86.4 |
| Height (cm) | 166.1±11.1 | | 163.6±8.0 |
| Weight (kg) | 76.0±15.9 | | 71.8±14.6 |
| Body Mass Index (kg/m^2^) | 27.4±4.8 | | 26.7±4.4 |
| Body Fat (%) | 33.1±7.3 | | 38.4±6.0 |
| Thigh Lean Mass (kg) | 5.1±1.0 | | 4.6±0.9 |
| Appendicular Lean Mass/height^2^ | 7.0±1.2 | | 6.3±1.0 |
| Isometric LE Strength (N-m) | 85.2±36.0 | | 73.2±25.5 |
| Relative Isokinetic LE Strength (N-m/kg) | 1.3±0.4 | | 1.2±0.2 |
| Neuromuscular Quality (N-m/kg) | 19.0±5.1 | | 18.7±3.5 |
| Handgrip Strength (kg) | 27.9±9.7 | | 25.6±5.7 |
| Moderate-to-Vigorous Activity (mins/week) | 112.5±57.9 | | 119.2±58.4 |
| Chair Rise Time (sec) | 11.0±3.5 | | 10.4±2.7 |
| Stair Climb Power (Watts) | 265.6±96.7 | | 265.3±67.7 |
| Six-Min Walk Gait Speed (m/sec) | 1.4±0.3 | | 1.3±0.2 |
| Charlson Index (% 10-yr survival) | 50.9±21.7 | | 57.6±16.5 |
| SPPB Score | 11.0±1.3 | | 11.3±1.0 |
| RBANS Score | 105.5±13.0 | | 109.3±12.1 |
|  |  |  | |
|  | **With Acceptable**  **Motor Unit Recordings**  **N=33** | | **With Unacceptable**  **Motor Unit Recordings**  **N=19** |
| Voluntary Activation (%) | 89.5±8.8 | | 90.7±7.0 |

LE= leg extensor; RBANS= repeatable battery for the assessment of neuropsychological status; SPPB= short physical performance battery.

| *Older Adults* | | | |  |
| --- | --- | --- | --- | --- |
|  | **With Acceptable**  **Motor Unit Recordings**  **N=43** | | **With Unacceptable**  **Motor Unit Recordings**  **N=22** | |
|  | **Males**  ***n*=23** | **Females**  ***n*=20** | **Males**  ***n*=3** | **Females**  ***n*=19** |
| Age (years) | 75.4±6.4 | 75.4±8.5 | 81.0±6.1 | 73.5±5.1 |
| Height (cm) | 173.6±7.6 | 157.4±7.5 | 175.2±11.0 | 161.8±5.9 |
| Weight (kg) | 84.1±12.9 | 66.6±14.1 | 87.3±16.8 | 69.3±13.0 |
| Body Mass Index (kg/m^2^) | 27.9±4.0 | 26.9±5.7 | 28.2±1.9 | 26.5±4.6 |
| Body Fat (%) | 29.2±5.7 | 37.5±6.5 | 32.0±2.3 | 39.4±5.8 |
| Thigh Lean Mass (kg) | 5.8±0.7 | 4.2±0.6 | 5.7±1.5 | 4.4±0.8 |
| Appendicular Lean Mass/height^2^ | 7.8±0.8 | 6.1±1.0 | 7.6±1.2 | 6.1±0.8 |
| Isometric LE Strength (N-m) | 109.6±32.3 | 63.5±17.9 | 116.7±27.7 | 69.8±14.7 |
| Relative Isokinetic LE Strength (N-m/kg) | 1.4±0.5 | 1.1±0.4 | 1.2±0.3 | 1.2±0.2 |
| Neuromuscular Quality (N-m/kg) | 20.2±5.4 | 17.7±4.6 | 19.25±3.9 | 18.6±3.5 |
| Handgrip Strength (kg) | 34.2±8.4 | 20.6±4.6 | 36.2±1.9 | 23.9±3.9 |
| Moderate-to-Vigorous Activity (mins/week) | 120.8±56.5 | 103.1±59.4 | 108.8±58.9 | 120.8±59.7 |
| Chair Rise Time (sec) | 10.6±2.3 | 11.4±4.6 | 12.1±2.7 | 10.2±2.6 |
| Stair Climb Power (Watts) | 313.2±84.0 | 210.8±81.4 | 269.3±55.4 | 264.7±70.8 |
| Six-Min Walk Gait Speed (m/sec) | 1.4±0.2 | 1.2±0.4 | 1.2±0.1 | 1.4±0.2 |
| Charlson Index (% 10-yr survival) | 50.0±22.3 | 51.9±21.5 | 53.4±0.0 | 58.3±17.7 |
| SPPB Score | 11.3±1.0 | 10.7±1.6 | 10.3±1.5 | 11.4±0.8 |
| RBANS Score | 102.6±13.2 | 108.8±12.3 | 99.3±20.6 | 110.8±10.3 |
|  | **With Acceptable**  **Motor Unit Recordings**  **N=33** | | **With Unacceptable**  **Motor Unit Recordings**  **N=19** | |
|  | **Males**  ***n*=16** | **Females**  ***n*=17** | **Males**  ***n*=3** | **Females**  ***n*=16** |
| Voluntary Activation (%) | 88.7±9.7 | 90.1±8.0 | 88.0±6.3 | 91.2±7.2 |

**Supplementary Table 2 (Disaggregated). Sex-Specific Descriptive Characteristics for Older Adults Possessing Acceptable and Unacceptable Trials for Motor Unit Recordings (mean ± SD).**
